# Supplementary material for: Effect of population inflow and outflow between rural and urban areas on regional antimicrobial use surveillance
Source: PLoS One. 2021 Mar 18;16(3):e0248338. doi: 10.1371/journal.pone.0248338 (PMC7971456; doi:10.1371/journal.pone.0248338)
Supplement: S1 Table — (DOCX) [file pone.0248338.s001.docx]

**S1 Table. Prefectures, secondary medical areas, and municipalities in Japan**

| **Prefecture** | **Secondary Medical Area** | **Municipality** |
| --- | --- | --- |
| Hokkaido | Minamioshima | Hakodate-shi |
|  |  | Hokuto-shi |
|  |  | Matsumaechou |
|  |  | Fukushimachou |
|  |  | Shiriuchichou |
|  |  | Kikonaichou |
|  |  | Nanaechou |
|  |  | Shikabechou |
|  |  | Morimachi |
|  | Minamihiyama | Esashichou |
|  |  | Kaminokunichou |
|  |  | Assabuchou |
|  |  | Otobechou |
|  |  | Okushirichou |
|  | Kitaoshimahiyama | Yakumochou |
|  |  | Oshamanbechou |
|  |  | Imakanechou |
|  |  | Setanachou |
|  | Sapporo | Sapporo-shi Chuou-ku |
|  |  | Sapporo-shi Kita-ku |
|  |  | Sapporo-shi Higashi-ku |
|  |  | Sapporo-shi Shiroishi-ku |
|  |  | Sapporo-shi Toyohira-ku |
|  |  | Sapporo-shi Minami-ku |
|  |  | Sapporo-shi Nishi-ku |
|  |  | Sapporo-shi Atsubetsu-ku |
|  |  | Sapporo-shi Teine-ku |
|  |  | Sapporo-shi Kiyota-ku |
|  |  | Ebetsu-shi |
|  |  | Chitose-shi |
|  |  | Eniwa-shi |
|  |  | Kitahiroshima-shi |
|  |  | Ishikari-shi |
|  |  | Toubetsuchou |
|  |  | Shinshinotsumura |
|  | Shiribeshi | Otaru-shi |
|  |  | Shimamakimura |
|  |  | Suttuchou |
|  |  | Kuromatsunaichou |
|  |  | Rankoshichou |
|  |  | Nisekochou |
|  |  | Makkarimura |
|  |  | Rusutsumura |
|  |  | Kimobetuchou |
|  |  | Kyougokuchou |
|  |  | Kucchanchou |
|  |  | Kyouwachou |
|  |  | Iwanaichou |
|  |  | Tomarimura |
|  |  | Kamoenaimura |
|  |  | Syakotanchou |
|  |  | Hurubirachou |
|  |  | Nikichou |
|  |  | Yoichichou |
|  |  | Akaigawamura |
|  | Minamisorachi | Yubari-shi |
|  |  | Iwamizawa-shi |
|  |  | Bibai-shi |
|  |  | Mikasa-shi |
|  |  | Nanporochou |
|  |  | Yunichou |
|  |  | Naganumachou |
|  |  | Kuriyamachou |
|  |  | Tsukigatachou |
|  | Nakasorachi | Ashibetsu-shi |
|  |  | Akabira-shi |
|  |  | Takikawa-shi |
|  |  | Sunagawa-shi |
|  |  | Utashinai-shi |
|  |  | Naiechou |
|  |  | Kamisunagawachou |
|  |  | Urausuchou |
|  |  | Shintotsukawachou |
|  |  | Uryuchou |
|  | Kitasorachi | Fukagawa-shi |
|  |  | Moseushichou |
|  |  | Chippubetuchou |
|  |  | Hokuryuchou |
|  |  | Numatachou |
|  | Nishiiburi | Muroran-shi |
|  |  | Noboribetsu-shi |
|  |  | Date-shi |
|  |  | Toyourachou |
|  |  | Soubetuchou |
|  |  | Touyakochou |
|  | Higashiiburi | Tomakomai-shi |
|  |  | Shiraoichou |
|  |  | Atsumachou |
|  |  | Abirachou |
|  |  | Mukawachou |
|  | Hidaka | Hidakachou |
|  |  | Biratorichou |
|  |  | Niikappuchou |
|  |  | Urakawachou |
|  |  | Samanichou |
|  |  | Erimochou |
|  |  | Shinhidakachou |
|  | Kamikawa-chubu | Asahikawa-shi |
|  |  | Takasuchou |
|  |  | Higashikagurachou |
|  |  | Toumachou |
|  |  | Pippuchou |
|  |  | Aibetsuchou |
|  |  | Kamikawachou |
|  |  | Higashikawachou |
|  |  | Bieichou |
|  |  | Horokanaichou |
|  | Kamikawa-hokubu | Shibetsu-shi |
|  |  | Nayoro-shi |
|  |  | Wassamuchou |
|  |  | Kenbuchichou |
|  |  | Shimokawachou |
|  |  | Bihukachou |
|  |  | Otoineppumura |
|  |  | Nakagawachou |
|  | Hurano | Furano-shi |
|  |  | Kamifuranochou |
|  |  | Nakafuranochou |
|  |  | Minamifuranochou |
|  |  | Shimukappumura |
|  | Rumoi | Rumoi-shi |
|  |  | Mashikechou |
|  |  | Obirachou |
|  |  | Tomamaechou |
|  |  | Haborochou |
|  |  | Syosanbetsumura |
|  |  | Enbetsuchou |
|  |  | Teshiochou |
|  | Souya | Wakkanai-shi |
|  |  | Sarufutsumura |
|  |  | Hamatonbetsuchou |
|  |  | Nakatonbetsuchou |
|  |  | Esashichou |
|  |  | Toyotomichou |
|  |  | Rebunchou |
|  |  | Rishirichou |
|  |  | Rishirifujichou |
|  |  | Horonobechou |
|  | Hokumou | Kitami-shi |
|  |  | Abashiri-shi |
|  |  | Bihorochou |
|  |  | Tsubetsuchou |
|  |  | Syarichou |
|  |  | Kiyosatochou |
|  |  | Koshimizuchou |
|  |  | Kuneppuchou |
|  |  | Oketochou |
|  |  | Oozorachou |
|  | Enmon | Mombetsu-shi |
|  |  | Saromachou |
|  |  | Engaruchou |
|  |  | Yubetuchou |
|  |  | Takinouechou |
|  |  | Okkopechou |
|  |  | Nishiokkopemura |
|  |  | Oumuchou |
|  | Tokachi | Obihiro-shi |
|  |  | Otofukechou |
|  |  | Shihorochou |
|  |  | Kamishihorochou |
|  |  | Shikaoichou |
|  |  | Shintokuchou |
|  |  | Shimizuchou |
|  |  | Memurochou |
|  |  | Nakasatsunaichou |
|  |  | Sarabetsumura |
|  |  | Taikichou |
|  |  | Hiroochou |
|  |  | Makubetsuchou |
|  |  | Ikedachou |
|  |  | Toyokorochou |
|  |  | Honbetsuchou |
|  |  | Asyorochou |
|  |  | Rikubetsuchou |
|  |  | Urahorochou |
|  | Kushiro | Kushiro-shi |
|  |  | Kushirochou |
|  |  | Akkeshichou |
|  |  | Hamanakachou |
|  |  | Shibechachou |
|  |  | Teshikagachou |
|  |  | Tsuruimura |
|  |  | Shiranukachou |
|  | Nemuro | Nemuro-shi |
|  |  | Betsukaichou |
|  |  | Nakashibetsuchou |
|  |  | Shibetsushou |
|  |  | Rausuchou |
| Aomori | Tsugaru-Chiiki | Hirosaki-shi |
|  |  | Kuroishi-shi |
|  |  | Hirakawa-shi |
|  |  | Nishimeyamura |
|  |  | Fujisakimachi |
|  |  | Owanimachi |
|  |  | Inakadatemura |
|  |  | Itayanagimachi |
|  | Hachinohe-Chiiki | Hachinohe-shi |
|  |  | Oirasechou |
|  |  | Sannnohemachi |
|  |  | Gonohemachi |
|  |  | Takkomachi |
|  |  | Nanbuchou |
|  |  | Hashikamichou |
|  |  | Shingoumura |
|  | Aomori-Chiiki | Aomori-shi |
|  |  | Hiranaimachi |
|  |  | Imabetsumachi |
|  |  | Yomogitamura |
|  |  | Sotogahamamachi |
|  | Seihokugo-Chiiki | Goshogawara-shi |
|  |  | Tsugaru-shi |
|  |  | Ajigasawamachi |
|  |  | Fukauramachi |
|  |  | Tsurutamachi |
|  |  | Nakadomarimachi |
|  | Kamitousan-Chiiki | Towada-shi |
|  |  | Misawa-shi |
|  |  | Nohejimachi |
|  |  | Shichinohemachi |
|  |  | Rokunohemachi |
|  |  | Yokohamamachi |
|  |  | Touhokumachi |
|  |  | Rokkashomura |
|  | Shimokita-Chiiki | Mutsu-shi |
|  |  | Oomamachi |
|  |  | Higashidoorimura |
|  |  | Kazamauramura |
|  |  | Saimura |
| Iwate | Morioka | Morioka-shi |
|  |  | Hachimantai-shi |
|  |  | Takizawa-shi |
|  |  | Shizukuishichou |
|  |  | Kuzumakimachi |
|  |  | Iwatemachi |
|  |  | Shiwachou |
|  |  | Yahabachou |
|  | Iwate-chubu | Hanamaki-shi |
|  |  | Kitakami-shi |
|  |  | Tono-shi |
|  |  | Nichiwagamachi |
|  | Tankou | Oshu-shi |
|  |  | Kanegasakichou |
|  | Ryoban | Ichinoseki-shi |
|  |  | Hiraizumichou |
|  | Kesen | Ofunato-shi |
|  |  | Rikuzentakata-shi |
|  |  | Sumitachou |
|  | Kamaichi | Kamaishi-shi |
|  |  | Otsuchichou |
|  | Miyako | Miyako-shi |
|  |  | Yamadamachi |
|  |  | Iwaizumichou |
|  |  | Tanohatamura |
|  | Kuji | Kuji-shi |
|  |  | Fudaimura |
|  |  | Nodamura |
|  |  | Hironochou |
|  | Ninohe | Ninohe-shi |
|  |  | Karumimachi |
|  |  | Kunohemura |
|  |  | Ichinohemachi |
| Miyagi | Sennan | Shiroishi-shi |
|  |  | Kakuda-shi |
|  |  | Zaoumachi |
|  |  | Shichikasyukumachi |
|  |  | Ogawaramachi |
|  |  | Muratamachi |
|  |  | Shibatamachi |
|  |  | Kawasakimachi |
|  |  | Marumorimachi |
|  | Sendai | Sendai-shi Aoba-ku |
|  |  | Sendai-shi Miyagino-ku |
|  |  | Sendai-shi Wakabayashi-ku |
|  |  | Sendai-shi Taihaku-ku |
|  |  | Sendai-shi Izumi-ku |
|  |  | Shiogama-shi |
|  |  | Natori-shi |
|  |  | Tagajo-shi |
|  |  | Iwanuma-shi |
|  |  | Tomiya-shi |
|  |  | Watarichou |
|  |  | Yamamotochou |
|  |  | Matsushimamachi |
|  |  | Shichigahamamachi |
|  |  | Rifuchou |
|  |  | Taiwachou |
|  |  | Osatochou |
|  |  | Ohiramura |
|  | Osaki, Kurihara | Kurihara-shi |
|  |  | Osaki-shi |
|  |  | Shikamachou |
|  |  | Kamimachi |
|  |  | Wakuyachou |
|  |  | Misatomachi |
|  | Ishinomaki, Tomeshi, Kesennuma | Ishinomaki-shi |
|  |  | Kesennuma-shi |
|  |  | Tome-shi |
|  |  | Higashimatsushima-shi |
|  |  | Onagawachou |
|  |  | Minamisanrikuchou |
| Akita | Odate, Kaduno | Odate-shi |
|  |  | Kazuno-shi |
|  |  | Kosakamachi |
|  | Kitaakita | Kitaakita-shi |
|  |  | Kamikoanimura |
|  | Noshiro, Yamamoto | Noshiro-shi |
|  |  | Fujisatomachi |
|  |  | Mitanechou |
|  |  | Happouchou |
|  | Akita-Shuhen | Akita-shi |
|  |  | Oga-shi |
|  |  | Katagami-shi |
|  |  | Gojomemachi |
|  |  | Hachirogatamachi |
|  |  | Ikawamachi |
|  |  | Ogatamura |
|  | Yurihonjou, Nikaho | Yurihonjo-shi |
|  |  | Nikaho-shi |
|  | Daisen, Senboku | Daisen-shi |
|  |  | Semboku-shi |
|  |  | Misatochou |
|  | Yokote | Yokote-shi |
|  | Yuzawa, Ogatsu | Yuzawa-shi |
|  |  | Ugomachi |
|  |  | Higashinarusemura |
| Yamagata | Murayama | Yamagata-shi |
|  |  | Sagae-shi |
|  |  | Kaminoyama-shi |
|  |  | Murayama-shi |
|  |  | Tendo-shi |
|  |  | Higashine-shi |
|  |  | Obanazawa-shi |
|  |  | Yamanobemachi |
|  |  | Nakayamamachi |
|  |  | Kahokuchou |
|  |  | Nishikawamachi |
|  |  | Asahimachi |
|  |  | Oemachi |
|  |  | Oishidamachi |
|  | Mogami | Shinjo-shi |
|  |  | Kaneyamamachi |
|  |  | Mogamimachi |
|  |  | Funagatamachi |
|  |  | Mamurogawamachi |
|  |  | Ohkuramura |
|  |  | Sakegawamura |
|  |  | Tozawamura |
|  | Okitama | Yonezawa-shi |
|  |  | Nagai-shi |
|  |  | Nanyo-shi |
|  |  | Takahatamachi |
|  |  | Kawanishimachi |
|  |  | Ogunimachi |
|  |  | Shiratakamachi |
|  |  | Iidemachi |
|  | Shonai | Tsuruoka-shi |
|  |  | Sakata-shi |
|  |  | Mikawamachi |
|  |  | Shonaimachi |
|  |  | Yuzamachi |
| Fukushima | Kenhoku | Fukushima-shi |
|  |  | Nihommatsu-shi |
|  |  | Date-shi |
|  |  | Motomiya-shi |
|  |  | Koorimachi |
|  |  | Kunimimachi |
|  |  | Kawamatamachi |
|  |  | Otamamura |
|  | Kennaka | Koriyama-shi |
|  |  | Sukagawa-shi |
|  |  | Tamura-shi |
|  |  | Kagamiishimachi |
|  |  | Teneimura |
|  |  | Ishikawamachi |
|  |  | Tamakawamura |
|  |  | Hiratamura |
|  |  | Asakawamachi |
|  |  | Furudonomachi |
|  |  | Miharumachi |
|  |  | Onomachi |
|  | Kennan | Shirakawa-shi |
|  |  | Nishigomura |
|  |  | Izumizakimura |
|  |  | Nakajimamura |
|  |  | Yabukimachi |
|  |  | Tanaguramachi |
|  |  | Yamatsurimachi |
|  |  | Hanawamachi |
|  |  | Samegawamura |
|  | Aizu | Aizuwakamatsu-shi |
|  |  | Kitakata-shi |
|  |  | Kitashiobaramura |
|  |  | Nishiaizumachi |
|  |  | Bandaimachi |
|  |  | Inawachiromachi |
|  |  | Aizubangemachi |
|  |  | Yugawamura |
|  |  | Yanaizumachi |
|  |  | Mishimamachi |
|  |  | Kaneyamamachi |
|  |  | Showamura |
|  |  | Aizumisatomachi |
|  | Minamiaizu | Shimogomachi |
|  |  | Hinoematamura |
|  |  | Tadamimachi |
|  |  | Minamiaizumachi |
|  | Soso | Soma-shi |
|  |  | Minamisoma-shi |
|  |  | Hironomachi |
|  |  | Narahamachi |
|  |  | Tomiokamachi |
|  |  | Kawauchimura |
|  |  | Okumamachi |
|  |  | Futabamachi |
|  |  | Namiemachi |
|  |  | Katsuraomura |
|  |  | Shinchimachi |
|  |  | Iitatemura |
|  | Iwaki | Iwakishi |
| Ibaraki | Mito | Mito-shi |
|  |  | Kasama-shi |
|  |  | Omitama-shi |
|  |  | Ibarakimachi |
|  |  | Oaraimachi |
|  |  | Shirosatomachi |
|  | Hitachi | Hitachi-shi |
|  |  | Takahagi-shi |
|  |  | Kitaibaraki-shi |
|  | Hitachioota, Hitachinaka | Hitachiota-shi |
|  |  | Hitachinaka-shi |
|  |  | Hitachiomiya-shi |
|  |  | Naka-shi |
|  |  | Tokaimura |
|  |  | Daigomachi |
|  | Rokkou | Kashima-shi |
|  |  | Itako-shi |
|  |  | Kamisu-shi |
|  |  | Namegata-shi |
|  |  | Hokota-shi |
|  | Tsuchiura | Tsuchiura-shi |
|  |  | Ishioka-shi |
|  |  | Kasumigaura-shi |
|  | Tsukuba | Joso-shi |
|  |  | Tsukuba-shi |
|  |  | Tsukubamirai-shi |
|  | Toride, Ryugasaki | Ryugasaki-shi |
|  |  | Toride-shi |
|  |  | Ushiku-shi |
|  |  | Moriya-shi |
|  |  | Inashiki-shi |
|  |  | Mihomura |
|  |  | Amimachi |
|  |  | Kawachimachi |
|  |  | Tonemachi |
|  | Chikusei, Simotsuma | Yuki-shi |
|  |  | Shimotsuma-shi |
|  |  | Chikusei-shi |
|  |  | Sakuragawa-shi |
|  |  | Yachiyomachi |
|  | Koga, Bandou | Koga-shi |
|  |  | Bando-shi |
|  |  | Gokamachi |
|  |  | Sakaimachi |
| Tochigi | Kenhoku | Otawara-shi |
|  |  | Yaita-shi |
|  |  | Nasushiobara-shi |
|  |  | Sakura-shi |
|  |  | Nasukarasuyama-shi |
|  |  | Shioyamachi |
|  |  | Takanezawamachi |
|  |  | Nasuma-shi |
|  |  | Nakagawamachi |
|  | Kensei | Kanuma-shi |
|  |  | Nikko-shi |
|  | Utsunomiya | Utsunomiya-shi |
|  | Kentou | Moka-shi |
|  |  | Mashikomachi |
|  |  | Motegimachi |
|  |  | Ischikaimachi |
|  |  | Hagamachi |
|  | Kennan | Tochigi-shi |
|  |  | Oyama-shi |
|  |  | Shimotsuke-shi |
|  |  | Kaminokawamachi |
|  |  | Mibumachi |
|  |  | Nogimachi |
|  | Ryomou | Ashikaga-shi |
|  |  | Sano-shi |
| Gunma | Maebashi | Maebashi-shi |
|  | Shibukawa | Shibukawa-shi |
|  |  | Shintomura |
|  |  | Yoshiokamachi |
|  | Isezaki | Isesaki-shi |
|  |  | Tamamuramachi |
|  | Takasaki, Annaka | Takasaki-shi |
|  |  | Annaka-shi |
|  | Fujioka | Fujioka-shi |
|  |  | Uenomura |
|  |  | Kannamachi |
|  | Tomioka | Tomioka-shi |
|  |  | Shimonitamachi |
|  |  | Nanmokumura |
|  |  | Kanramachi |
|  | Agatuma | Nakanojomachi |
|  |  | Naganoharamachi |
|  |  | Tsumagoimura |
|  |  | Kusatsumachi |
|  |  | Takayamamura |
|  |  | Higashiagatsumamachi |
|  | Numata | Numata-shi |
|  |  | Katanashimura |
|  |  | Kawabamura |
|  |  | Showamura |
|  |  | Minakamimachi |
|  | Kiryu | Kiryu-shi |
|  |  | Midori-shi |
|  | Ota, Tatebayashi | Ota-shi |
|  |  | Tatebayashi-shi |
|  |  | Itakuramachi |
|  |  | Meiwamachi |
|  |  | Chiyodamachi |
|  |  | Oizumimachi |
|  |  | Oramachi |
| Saitama | Nanbu | Kawaguchi-shi |
|  |  | Warabi-shi |
|  |  | Toda-shi |
|  | Nanseibu | Asaka-shi |
|  |  | Shiki-shi |
|  |  | Wako-shi |
|  |  | Niiza-shi |
|  |  | Fujimi-shi |
|  |  | Fujimino-shi |
|  |  | Miyoshimachi |
|  | Toubu | Kasukabe-shi |
|  |  | Soka-shi |
|  |  | Koshigaya-shi |
|  |  | Yashio-shi |
|  |  | Misato-shi |
|  |  | Yoshikawa-shi |
|  |  | Matsubushimachi |
|  | Saitama | Saitama-shi Nishi-ku |
|  |  | Saitama-shi Kita-ku |
|  |  | Saitama-shi Omiya-ku |
|  |  | Saitama-shi Minuma-ku |
|  |  | Saitama-shi Chuou-ku |
|  |  | Saitama-shi Sakuramach |
|  |  | Saitama-shi Urawa-ku |
|  |  | Saitama-shi Minami-ku |
|  |  | Saitama-shi Midori-ku |
|  |  | Saitama-shi Iwatsuki-ku |
|  | Kenou | Konosu-shi |
|  |  | Ageo-shi |
|  |  | Okegawa-shi |
|  |  | Kitamoto-shi |
|  |  | Inamachi |
|  | Kawagoehiki | Kawagoe-shi |
|  |  | Higashimatsuyama-shi |
|  |  | Sakado-shi |
|  |  | Tsurugashima-shi |
|  |  | Moroyamamachi |
|  |  | Ogosemachi |
|  |  | Namegawamachi |
|  |  | Ranzanmachi |
|  |  | Ogawamachi |
|  |  | Kawajimamachi |
|  |  | Yoshimimachi |
|  |  | Hatoyamamachi |
|  |  | Tokigawamachi |
|  |  | Higashichichibumura |
|  | Seibu | Tokorozawa-shi |
|  |  | Hanno-shi |
|  |  | Sayama-shi |
|  |  | Iruma-shi |
|  |  | Hidaka-shi |
|  | Tone | Gyoda-shi |
|  |  | Kazo-shi |
|  |  | Hanyu-shi |
|  |  | Kuki-shi |
|  |  | Hasuda-shi |
|  |  | Satte-shi |
|  |  | Miyashiromachi |
|  |  | Shiraoka-shi |
|  |  | Sugitomachi |
|  | Hokubu | Kumagaya-shi |
|  |  | Honjo-shi |
|  |  | Fukaya-shi |
|  |  | Misatomachi |
|  |  | Kamikawamachi |
|  |  | Kamisatomachi |
|  |  | Yoriimachi |
|  | Chichibu | Chichibu-shi |
|  |  | Yokozemachi |
|  |  | Minanomachi |
|  |  | Nagatoromachi |
|  |  | Oganomachi |
| Chiba | Chiba | Chiba-shi Chuou-ku |
|  |  | Chiba-shi Hanamigawa-ku |
|  |  | Chiba-shi Inage-ku |
|  |  | Chiba-shi Wakaba-ku |
|  |  | Chiba-shi Midori-ku |
|  |  | Chiba-shi Mihama-ku |
|  | Toukatsu-nanbu | Ichikawa-shi |
|  |  | Funabashi-shi |
|  |  | Narashino-shi |
|  |  | Yachiyo-shi |
|  |  | Kamagaya-shi |
|  |  | Urayasu-shi |
|  | Toukatsu-hokubu | Matsudo-shi |
|  |  | Noda-shi |
|  |  | Kashiwa-shi |
|  |  | Nagareyama-shi |
|  |  | Abiko-shi |
|  | Inba | Narita-shi |
|  |  | Sakura-shi |
|  |  | Yotsukaido-shi |
|  |  | Yachimata-shi |
|  |  | Inzai-shi |
|  |  | Shiroi-shi |
|  |  | Tomisato-shi |
|  |  | Shisuimachi |
|  |  | Sakaemachi |
|  | Katorikaisou | Choshi-shi |
|  |  | Asahi-shi |
|  |  | Sosa-shi |
|  |  | Katori-shi |
|  |  | Kouzakimachi |
|  |  | Takomachi |
|  |  | Tohnoshoma-shi |
|  | Sanmushichouseiisumi | Mobara-shi |
|  |  | Togane-shi |
|  |  | Katsura-shi |
|  |  | Sammu-shi |
|  |  | Isumi-shi |
|  |  | Oamishirasato-shi |
|  |  | Kujukurimachi |
|  |  | Shibayamamachi |
|  |  | Yokobahikarimachi |
|  |  | Ichinomiyamachi |
|  |  | Mutsuzawamachi |
|  |  | Choseimura |
|  |  | Shirakomachi |
|  |  | Nagaramachi |
|  |  | Chonanmachi |
|  |  | Otakimachi |
|  |  | Onjukumachi |
|  | Awa | Tateyama-shi |
|  |  | Kamogawa-shi |
|  |  | Minamiboso-shi |
|  |  | Kyonanmachi |
|  | Kimitsu | Kisarazu-shi |
|  |  | Kimitsu-shi |
|  |  | Futtsu-shi |
|  |  | Sodegaura-shi |
|  | Ichihara | Ichihara-shi |
| Tokyo | Ku-chuoubu | Chiyoda-ku |
|  |  | Chuo-ku |
|  |  | Minato-ku |
|  |  | Bunkyo-ku |
|  |  | Taito-ku |
|  | Ku-nanbu | Shinagawa-ku |
|  |  | Ota-ku |
|  | Ku-seinanbu | Meguro-ku |
|  |  | Setagayaku |
|  |  | Shibuya-ku |
|  | Ku-seibu | Shinjuku-ku |
|  |  | Nakano-ku |
|  |  | Suginami-ku |
|  | Ku-seihokubu | Toshima-ku |
|  |  | Kita-ku |
|  |  | Itabashi-ku |
|  |  | Nerima-ku |
|  | Ku-touhokubu | Arakawa-ku |
|  |  | Adachi-ku |
|  |  | Katsushika-ku |
|  | Ku-toubu | Sumida-ku |
|  |  | Koto-ku |
|  |  | Edogawa-ku |
|  | Nishitama | Ome-shi |
|  |  | Fussa-shi |
|  |  | Hamura-shi |
|  |  | Akiruno-shi |
|  |  | Mizuhomachi |
|  |  | Hinodemachi |
|  |  | Hinoharamura |
|  |  | Okutamamachi |
|  | Minamitama | Hachioji-shi |
|  |  | Machida-shi |
|  |  | Hino-shi |
|  |  | Tama-shi |
|  |  | Inagi-shi |
|  | Kitatama-seibu | Tachikawa-shi |
|  |  | Akishima-shi |
|  |  | Kokubunji-shi |
|  |  | Kunitachi-shi |
|  |  | Higashiyamato-shi |
|  |  | Musashimurayama-shi |
|  | Kitatama-nanbu | Musashino-shi |
|  |  | Mitaka-shi |
|  |  | Fuchu-shi |
|  |  | Chofu-shi |
|  |  | Koganei-shi |
|  |  | Komae-shi |
|  | Kitatama-hokubu | Kodaira-shi |
|  |  | Higashimurayama-shi |
|  |  | Kiyose-shi |
|  |  | Higashikurume-shi |
|  |  | Nishitokyo-shi |
|  | Tousyo | Oshimamachi |
|  |  | Toshimamura |
|  |  | Niijimamura |
|  |  | Kozushimamura |
|  |  | Miyakemura |
|  |  | Mikurajimamura |
|  |  | Hachijomachi |
|  |  | Aogashimamura |
|  |  | Ogasawaramura |
| Kanagawa | Yokohama-hokubu | Yokohama-shi Tsurumi-ku |
|  |  | Yokohama-shi Kanagawa-ku |
|  |  | Yokohama-shi Kouhoku-ku |
|  |  | Yokohama-shi Midori-ku |
|  |  | Yokohama-shi Aoba-ku |
|  |  | Yokohama-shi Tsuzuki-ku |
|  | Yokohama-seibu | Yokohama-shi Nishi-ku |
|  |  | Yokohama-shi Hodogaya-ku |
|  |  | Yokohama-shi Totsuka-ku |
|  |  | Yokohama-shi Asahi-ku |
|  |  | Yokohama-shi Seya-ku |
|  |  | Yokohama-shi Izumi-ku |
|  | Yokohama-nanbu | Yokohama-shi Naka-ku |
|  |  | Yokohama-shi Minami-ku |
|  |  | Yokohama-shi Isogo-ku |
|  |  | Yokohama-shi Kanazawa-ku |
|  |  | Yokohama-shi Kounan-ku |
|  |  | Yokohama-shi Sakae-ku |
|  | Kawasaki-hokubu | Kawasaki-shi Takatsu-ku |
|  |  | Kawasaki-shi Tama-ku |
|  |  | Kawasaki-shi Miyamae-ku |
|  |  | Kawasaki-shi Asao-ku |
|  | Kawasaki-nanbu | Kawasaki-shi Kawasaki-ku |
|  |  | Kawasaki-shi Saiwai-ku |
|  |  | Kawasaki-shi Nakahara-ku |
|  | Yokosuka, Miura | Yokosuka-shi |
|  |  | Kamakura-shi |
|  |  | Zushi-shi |
|  |  | Miura-shi |
|  |  | Hayamachou |
|  | Shonan-toubu | Fujisawa-shi |
|  |  | Chigasaki-shi |
|  |  | Samukawachou |
|  | Shonan-seibu | Hiratsuka-shi |
|  |  | Hadano-shi |
|  |  | Isehara-shi |
|  |  | Oisomachi |
|  |  | Ninomiyamachi |
|  | Kenou | Atsugi-shi |
|  |  | Yamato-shi |
|  |  | Ebina-shi |
|  |  | Zama-shi |
|  |  | Ayase-shi |
|  |  | Aikawamachi |
|  |  | Kiyokawamura |
|  | Sagamihara | Sagamihara-shi Midoriku |
|  |  | Sagamihara-shi Chuouku |
|  |  | Sagamihara-shi Minamiku |
|  | Kensei | Odawara-shi |
|  |  | Minamiashigara-shi |
|  |  | Nakaimachi |
|  |  | Oimachi |
|  |  | Matsudamachi |
|  |  | Yamakitamachi |
|  |  | Kaiseimachi |
|  |  | Hakonemachi |
|  |  | Manazurumachi |
|  |  | Yugawaramachi |
| Niigata | Kaetsu | Shibata-shi |
|  |  | Murakami-shi |
|  |  | Tainai-shi |
|  |  | Seiroumachi |
|  |  | Sekikawamura |
|  |  | Awashimauramura |
|  | Niigata | Niiigata-shi Kita-ku |
|  |  | Niiigata-shi Higashi-ku |
|  |  | Niiigata-shi Chuou-ku |
|  |  | Niiigata-shi Kounan-ku |
|  |  | Niiigata-shi Akiha-ku |
|  |  | Niiigata-shi Minami-ku |
|  |  | Niiigata-shi Nishi-ku |
|  |  | Niiigata-shi Nishikan-ku |
|  |  | Gosen-shi |
|  |  | Agano-shi |
|  |  | Agamachi |
|  | Kenou | Sanjo-shi |
|  |  | Kamo-shi |
|  |  | Tsubame-shi |
|  |  | Yahikomura |
|  |  | Tagamimachi |
|  | Chuetsu | Nagaoka-shi |
|  |  | Kashiwazaki-shi |
|  |  | Ojiya-shi |
|  |  | Mitsuke-shi |
|  |  | Izumozakimachi |
|  |  | Kariwamura |
|  | Uonuma | Tokamachi-shi |
|  |  | Uonuma-shi |
|  |  | Minamiuonuma-shi |
|  |  | Yuzawamachi |
|  |  | Tsunanmachi |
|  | Jouetsu | Itoigawa-shi |
|  |  | Myoko-shi |
|  |  | Joetsu-shi |
|  | Sado | Sado-shi |
| Toyama | Nikawa | Uozu-shi |
|  |  | Kurobe-shi |
|  |  | Nyuzenmachi |
|  |  | Asahimachi |
|  | Toyama | Toyama-shi |
|  |  | Namerikawa-shi |
|  |  | Funahashimura |
|  |  | Kamiichimachi |
|  |  | Tateyamamachi |
|  | Takaoka | Takaoka-shi |
|  |  | Himi-shi |
|  |  | Imizu-shi |
|  | Tonami | Tonami-shi |
|  |  | Oyabe-shi |
|  |  | Nanto-shi |
| Ishikawa | Minamikaga | Komatsu-shi |
|  |  | Kaga-shi |
|  |  | Nomi-shi |
|  |  | Kawakitamachi |
|  | Ishikawa-chuou | Kanazawa-shi |
|  |  | Kahoku-shi |
|  |  | Hakusan-shi |
|  |  | Nonoichi-shi |
|  |  | Tsubatamachi |
|  |  | Uchinadamachi |
|  | Noto-chubu | Nanao-shi |
|  |  | Hakui-shi |
|  |  | Shikamachi |
|  |  | Houdatsushimizuchou |
|  |  | Nakanotomachi |
|  | Noto-hokubu | Wajima-shi |
|  |  | Suzu-shi |
|  |  | Anamizumachi |
|  |  | Notochou |
| Fukui | Fukui, Sakai | Fukui-shi |
|  |  | Awara-shi |
|  |  | Sakai-shi |
|  |  | Eiheijichou |
|  | Okuetsu | Ono-shi |
|  |  | Katsuyama-shi |
|  | Tannan | Sabae-shi |
|  |  | Echizen-shi |
|  |  | Ikedachou |
|  |  | Minamiechizenchou |
|  |  | Echizenchou |
|  | Reinan | Tsuruga-shi |
|  |  | Obama-shi |
|  |  | Mihamachou |
|  |  | Takahamachou |
|  |  | Aoichou |
|  |  | Wakasachou |
| Yamanashi | Chuhoku | Kofu-shi |
|  |  | Nirasaki-shi |
|  |  | Minamiarupusu-shi |
|  |  | Hokuto-shi |
|  |  | Kai-shi |
|  |  | Chuo-shi |
|  |  | Showachou |
|  | Kyoutou | Yamanashi-shi |
|  |  | Fuefuki-shi |
|  |  | Koshu-shi |
|  | Kyonan | Ichikawamisatochou |
|  |  | Hayakawachou |
|  |  | Minobuchou |
|  |  | Nanbuchou |
|  |  | Fujikawamachi |
|  | Fuji, Toubu | Fujiyoshida-shi |
|  |  | Tsuru-shi |
|  |  | Otsuki-shi |
|  |  | Uenohara-shi |
|  |  | Doushimura |
|  |  | Nishikatsurachou |
|  |  | Oshinomura |
|  |  | Yamanakakomura |
|  |  | Narusawamura |
|  |  | Fujikawaguchikomachi |
|  |  | Kosugemura |
|  |  | Tabayamamura |
| Nagano | Saku | Komoro-shi |
|  |  | Saku-shi |
|  |  | Koumimachi |
|  |  | Kawakamimura |
|  |  | Minamimakimura |
|  |  | Minamiaikimura |
|  |  | Kitaaikimura |
|  |  | Sakuhomachi |
|  |  | Karuizawamachi |
|  |  | Miyotamachi |
|  |  | Tateshinamachi |
|  | Kamiko | Ueda-shi |
|  |  | Tomi-shi |
|  |  | Aokimura |
|  |  | Nagawamachi |
|  | Suwa | Okaya-shi |
|  |  | Suwa-shi |
|  |  | Chino-shi |
|  |  | Shimosuwamachi |
|  |  | Fujimimachi |
|  |  | Haramura |
|  | Kamiina | Ina-shi |
|  |  | Komagane-shi |
|  |  | Tatsunomachi |
|  |  | Minowamachi |
|  |  | Iijimamachi |
|  |  | Minamiminowamura |
|  |  | Nakagawamura |
|  |  | Miyatamura |
|  | Hanni | Iida-shi |
|  |  | Matsukawamachi |
|  |  | Takamorimachi |
|  |  | Ananchou |
|  |  | Achimura |
|  |  | Hirayamura |
|  |  | Nebamura |
|  |  | Shimojomura |
|  |  | Urugimura |
|  |  | Tenryumura |
|  |  | Yasuokamura |
|  |  | Takagimura |
|  |  | Toyookamura |
|  |  | Ooshikamura |
|  | Kiso | Agematsumachi |
|  |  | Nagisomachi |
|  |  | Kisomura |
|  |  | Otakimura |
|  |  | Ookuwamura |
|  |  | Kisomachi |
|  | Matsumoto | Matsumoto-shi |
|  |  | Shiojiri-shi |
|  |  | Azumino-shi |
|  |  | Omimura |
|  |  | Ikusakamura |
|  |  | Yamagatamura |
|  |  | Asahimura |
|  |  | Chikuhokumura |
|  | Taihoku | Omachi-shi |
|  |  | Ikedachou |
|  |  | Matsukawamura |
|  |  | Hakubamura |
|  |  | Otarimura |
|  | Nagano | Nagano-shi |
|  |  | Suzaka-shi |
|  |  | Chikuma-shi |
|  |  | Sakakimachi |
|  |  | Obusemachi |
|  |  | Takayamamura |
|  |  | Shinanomachi |
|  |  | Ogawamura |
|  |  | Iizunamachi |
|  | Hokushin | Nakano-shi |
|  |  | Iiyama-shi |
|  |  | Yamanouchimachi |
|  |  | Kijimadairamura |
|  |  | Nozawaonsenmura |
|  |  | Sakaemura |
| Gifu | Gifu | Gifu-shi |
|  |  | Hashima-shi |
|  |  | Kakamigahara-shi |
|  |  | Yamagata-shi |
|  |  | Mizuho-shi |
|  |  | Motosu-shi |
|  |  | Ginanchou |
|  |  | Kasamatsuchou |
|  |  | Kitagatachou |
|  | Seinou | Ogaki-shi |
|  |  | Kaizu-shi |
|  |  | Yorochou |
|  |  | Taruichou |
|  |  | Sekigaharachou |
|  |  | Godochou |
|  |  | Wanouchichou |
|  |  | Anpachichou |
|  |  | Ibigawachou |
|  |  | Onochou |
|  |  | Ikedachou |
|  | Chunou | Seki-shi |
|  |  | Mino-shi |
|  |  | Minokamo-shi |
|  |  | Kani-shi |
|  |  | Gujo-shi |
|  |  | Sakahogichou |
|  |  | Tomikachou |
|  |  | Kawabechou |
|  |  | Hichisochou |
|  |  | Yaotsumachi |
|  |  | Shirakawachou |
|  |  | Higashishirakawachou |
|  |  | Mitakechou |
|  | Tounou | Tajimi-shi |
|  |  | Nakatsugawa-shi |
|  |  | Mizunami-shi |
|  |  | Ena-shi |
|  |  | Toki-shi |
|  | Hida | Takayama-shi |
|  |  | Hida-shi |
|  |  | Gero-shi |
|  |  | Shiarakawamura |
| Shizuoka | Kamo | Shimoda-shi |
|  |  | Higashiizuchou |
|  |  | Kawazuchou |
|  |  | Minamiizuchou |
|  |  | Matsuzakichou |
|  |  | Nishiizuchou |
|  | Atamiitou | Atami-shi |
|  |  | Ito-shi |
|  | Suntoutagata | Numazu-shi |
|  |  | Mishima-shi |
|  |  | Gotemba-shi |
|  |  | Susono-shi |
|  |  | Izu-shi |
|  |  | Izunokuni-shi |
|  |  | Kannamichou |
|  |  | Shimizuchou |
|  |  | Nagaizumichou |
|  |  | Oyamachou |
|  | Fuji | Fujinomiya-shi |
|  |  | Fuji-shi |
|  | Shizuoka | Shizuoka-shi Aoi-ku |
|  |  | Shizuoka-shi Suruga-ku |
|  |  | Shizuoka-shi Shimizu-ku |
|  | Shidahaibara | Shimada-shi |
|  |  | Yaizushi |
|  |  | Fujieda-shi |
|  |  | Makinohara-shi |
|  |  | Yoshidachou |
|  |  | Kawanehonchou |
|  | Chutouen | Iwata-shi |
|  |  | Kakegawa-shi |
|  |  | Fukuroi-shi |
|  |  | Omaezaki-shi |
|  |  | Kikugawa-shi |
|  |  | Morimachi |
|  | Seibu | Hamamatsu-shi Naka-ku |
|  |  | Hamamatsu-shi Higashi-ku |
|  |  | Hamamatsu-shi Nishi-ku |
|  |  | Hamamatsu-shi Minami-ku |
|  |  | Hamamatsu-shi Kita-ku |
|  |  | Hamamatsu-shi Hamakita-ku |
|  |  | Hamamatsu-shi Tenryu-ku |
|  |  | Kosai-shi |
| Aichi | Nagoya | Nagoya-shi Chikusa-ku |
|  |  | Nagoya-shi Higashi-ku |
|  |  | Nagoya-shi Kita-ku |
|  |  | Nagoya-shi Nishi-ku |
|  |  | Nagoya-shi Nakamura-ku |
|  |  | Nagoya-shi Naka-ku |
|  |  | Nagoya-shi Showa-ku |
|  |  | Nagoya-shi Mizuho-ku |
|  |  | Nagoya-shi Atsuta-ku |
|  |  | Nagoya-shi Nakagawa-ku |
|  |  | Nagoya-shi Minato-ku |
|  |  | Nagoya-shi Minami-ku |
|  |  | Nagoya-shi Moriyama-ku |
|  |  | Nagoya-shi Midori-ku |
|  |  | Nagoya-shi Meito-ku |
|  |  | Nagoya-shi Tenpaku-ku |
|  | Ama | Tsushima-shi |
|  |  | Aisai-shi |
|  |  | Yatomi-shi |
|  |  | Ama-shi |
|  |  | Oharuchou |
|  |  | Kaniechou |
|  |  | Tobishimamura |
|  | Owari-chubu | Kiyosu-shi |
|  |  | Kitanagoya-shi |
|  |  | Toyoyamachou |
|  | Owari-toubu | Seto-shi |
|  |  | Owariasahi-shi |
|  |  | Toyoake-shi |
|  |  | Nisshin-shi |
|  |  | Nagakute-shi |
|  |  | Togochou |
|  | Owari-seibu | Ichinomiya-shi |
|  |  | Inazawa-shi |
|  | Owari-hokubu | Kasugai-shi |
|  |  | Inuyama-shi |
|  |  | Konan-shi |
|  |  | Komaki-shi |
|  |  | Iwakura-shi |
|  |  | Oguchichou |
|  |  | Fusochou |
|  | Chitahantou | Handa-shi |
|  |  | Tokoname-shi |
|  |  | Tokai-shi |
|  |  | Obu-shi |
|  |  | Chita-shi |
|  |  | Aguichou |
|  |  | Higashiurachou |
|  |  | Minamichitachou |
|  |  | Mihamachou |
|  |  | Taketoyochou |
|  | Nishimikawa-hokubu | Toyota-shi |
|  |  | Miyoshi-shi |
|  | Nishimikawa-nanbunishi | Hekinan-shi |
|  |  | Kariya-shi |
|  |  | Anjo-shi |
|  |  | Nishio-shi |
|  |  | Chiryu-shi |
|  |  | Takahama-shi |
|  | Nishimikawa-nanbuhigashi | Okazaki-shi |
|  |  | Kotachou |
|  | Higashimikawa-hokubu | Shinshiro-shi |
|  |  | Shitarachou |
|  |  | Toeichou |
|  |  | Toyonemura |
|  | Higashimikawa-nanbu | Toyohash-ishi |
|  |  | Toyokawa-shi |
|  |  | Gamagori-shi |
|  |  | Tahara-shi |
| Mie | Hokusei | Yokkaichi-shi |
|  |  | Kuwana-shi |
|  |  | Suzuka-shi |
|  |  | Kameyama-shi |
|  |  | Inabe-shi |
|  |  | Kisosakichou |
|  |  | Toinchou |
|  |  | Komonochou |
|  |  | Asahichou |
|  |  | Kawagoechou |
|  | Chusei-iga | Tsu-shi |
|  |  | Nabari-shi |
|  |  | Iga-shi |
|  | Nansei-shima | Ise-shi |
|  |  | Matsusaka-shi |
|  |  | Toba-shi |
|  |  | Shima-shi |
|  |  | Takichou |
|  |  | Meiwachou |
|  |  | Odaichou |
|  |  | Tamakichou |
|  |  | Wataraichou |
|  |  | Taikichou |
|  |  | Minamiisechou |
|  | Higashikishuu | Owase-shi |
|  |  | Kumano-shi |
|  |  | Kihokuchou |
|  |  | Mihamachou |
|  |  | Kihochou |
| Shiga | Otsu | Otsu-shi |
|  | Konan | Kusatsu-shi |
|  |  | Moriyama-shi |
|  |  | Ritto-shi |
|  |  | Yasu-shi |
|  | Kouga | Koka-shi |
|  |  | Konan-shi |
|  | Higashioumi | Omihachiman-shi |
|  |  | Higashiomi-shi |
|  |  | Hinochou |
|  |  | Ryuohchou |
|  | Kotou | Hikone-shi |
|  |  | Aishochou |
|  |  | Toyosatochou |
|  |  | Kourachou |
|  |  | Tagachou |
|  | Kohoku | Nagahama-shi |
|  |  | Maibara-shi |
|  | Kosai | Takashima-shi |
| Kyoto | Tango | Miyazu-shi |
|  |  | Kyotango-shi |
|  |  | Inechou |
|  |  | Yosanochou |
|  | Chutan | Fukuchiyama-shi |
|  |  | Maizuru-shi |
|  |  | Ayabe-shi |
|  | Nantan | Kameoka-shi |
|  |  | Nantan-shi |
|  |  | Kyotambachou |
|  | Kyoto, Otokuni | Kyoto-shi Kita-ku |
|  |  | Kyoto-shi Kamigyo-ku |
|  |  | Kyoto-shi Sakyo-ku |
|  |  | Kyoto-shi Nakagyo-ku |
|  |  | Kyoto-shi Higashiyama-ku |
|  |  | Kyoto-shi Shimogyo-ku |
|  |  | Kyoto-shi Minami-ku |
|  |  | Kyoto-shi Ukyo-ku |
|  |  | Kyoto-shi Fushimi-ku |
|  |  | Kyoto-shi Yamashina-ku |
|  |  | Kyoto-shi Nishikyo-ku |
|  |  | Muko-shi |
|  |  | Nagaokakyo-shi |
|  |  | Oyamazakichou |
|  | Sanjo-kita | Uji-shi |
|  |  | Joyo-shi |
|  |  | Yawata-shi |
|  |  | Kyotanabe-shi |
|  |  | Kumiyamacho |
|  |  | Idechou |
|  |  | Ujitawarachou |
|  | Sanjo-minami | Kizugawa-shi |
|  |  | Kasagichou |
|  |  | Wazukachou |
|  |  | Seikachou |
|  |  | Minamiyamashiromura |
| Osaka | Toyono | Toyonaka-shi |
|  |  | Ikeda-shi |
|  |  | Suita-shi |
|  |  | Mino-shi |
|  |  | Toyonochou |
|  |  | Nosechou |
|  | Mishima | Takatsuki-shi |
|  |  | Ibaraki-shi |
|  |  | Settsu-shi |
|  |  | Shimamotochou |
|  | Kitakawachi | Moriguchi-shi |
|  |  | Hirakata-shi |
|  |  | Neyagawa-shi |
|  |  | Daito-shi |
|  |  | Kadoma-shi |
|  |  | Shijonawate-shi |
|  |  | Katano-shi |
|  | Nakakawachi | Yao-shi |
|  |  | Kashiwara-shi |
|  |  | Higashiosaka-shi |
|  | Minamikawachi | Tondabayashi-shi |
|  |  | Kawachinagano-shi |
|  |  | Matsubara-shi |
|  |  | Habikino-shi |
|  |  | Fujiidera-shi |
|  |  | Osakasayama-shi |
|  |  | Taishichou |
|  |  | Kananchou |
|  |  | Chihayaakasakamura |
|  | Sakai-shi | Sakai-shi Sakai-ku |
|  |  | Sakai-shi Nakai-ku |
|  |  | Sakai-shi Higash-ku |
|  |  | Sakai-shi Nishi-ku |
|  |  | Sakai-shi Minami-ku |
|  |  | Sakai-shi Kita-ku |
|  |  | Sakai-shi Mihara-ku |
|  | Senshu | Kishiwada-shi |
|  |  | Izumiotsu-shi |
|  |  | Kaizuka-shi |
|  |  | Izumisano-shi |
|  |  | Izumi-shi |
|  |  | Takaishi-shi |
|  |  | Sennan-shi |
|  |  | Hannan-shi |
|  |  | Tadaokachou |
|  |  | Kumatorichou |
|  |  | Tajirichou |
|  |  | Misakichou |
|  | Osaka-shi | Osaka-shi Miyakojima-ku |
|  |  | Osaka-shi Fukushima-ku |
|  |  | Osaka-shi Konohana-ku |
|  |  | Osaka-shi Nishi-ku |
|  |  | Osaka-shi Minato-ku |
|  |  | Osaka-shi Taisho-ku |
|  |  | Osaka-shi Tennoji-ku |
|  |  | Osaka-shi Naniwa-ku |
|  |  | Osaka-shi Nishiyodogawa-ku |
|  |  | Osaka-shi Higashiyodogawa-ku |
|  |  | Osaka-shi Higashinari-ku |
|  |  | Osaka-shi Ikuno-ku |
|  |  | Osaka-shi Asahi-ku |
|  |  | Osaka-shi Joto-ku |
|  |  | Osaka-shi Abeno-ku |
|  |  | Osaka-shi Sumiyoshi-ku |
|  |  | Osaka-shi Higashisumiyoshi-ku |
|  |  | Osaka-shi Nishinari-ku |
|  |  | Osaka-shi Yodogawa-ku |
|  |  | Osaka-shi Tsurumi-ku |
|  |  | Osaka-shi Suminoe-ku |
|  |  | Osaka-shi Hirano-ku |
|  |  | Osaka-shi Kita-ku |
|  |  | Osaka-shi Chuou-ku |
| Hyogo | Kobe | Kobe-shi Higashinada-ku |
|  |  | Kobe-shi Nada-ku |
|  |  | Kobe-shi Hyogo-ku |
|  |  | Kobe-shi Nagata-ku |
|  |  | Kobe-shi Suma-ku |
|  |  | Kobe-shi Tarumi-ku |
|  |  | Kobe-shi Kita-ku |
|  |  | Kobe-shi Chuou-ku |
|  |  | Kobe-shi Nishi-ku |
|  | Hanshin-minami | Amagasaki-shi |
|  |  | Nishinomiya-shi |
|  |  | Ashiya-shi |
|  | Hanshin-kita | Itami-shi |
|  |  | Takarazuka-shi |
|  |  | Kawanishi-shi |
|  |  | Sanda-shi |
|  |  | Inagawachou |
|  | Higashiharima | Akashi-shi |
|  |  | Kakogawa-shi |
|  |  | Takasago-shi |
|  |  | Inamichou |
|  |  | Harimachou |
|  | Kitaharima | Nishiwaki-shi |
|  |  | Miki-shi |
|  |  | Ono-shi |
|  |  | Kasai-shi |
|  |  | Kato-shi |
|  |  | Takachou |
|  | Nakaharima | Himeji-shi |
|  |  | Ichikawachou |
|  |  | Fukusakichou |
|  |  | Kamikawachou |
|  | Nishiharima | Aioi-shi |
|  |  | Ako-shi |
|  |  | Shiso-shi |
|  |  | Tatsunoshi |
|  |  | Taishichou |
|  |  | Kamigorichou |
|  |  | Sayochou |
|  | Tajima | Toyoka-shi |
|  |  | Yabu-shi |
|  |  | Asago-shi |
|  |  | Kamichou |
|  |  | Shinonsenchou |
|  | Tanba | Tanbasasayama-shi |
|  |  | Tamba-shi |
|  | Awaji | Sumoto-shi |
|  |  | Minamiawaji-shi |
|  |  | Awaji-shi |
| Nara | Nara | Nara-shi |
|  | Touwa | Tenri-shi |
|  |  | Sakurai-shi |
|  |  | Uda-shi |
|  |  | Yamazoemura |
|  |  | Kawanishimachi |
|  |  | Miyakechou |
|  |  | Tawaramotochou |
|  |  | Sonimura |
|  |  | Mitsuemura |
|  | Seiwa | Yamatokoriyama-shi |
|  |  | Ikoma-shi |
|  |  | Hegurichou |
|  |  | Sangochou |
|  |  | Ikarugachou |
|  |  | Andochou |
|  |  | Kanmakichou |
|  |  | Ojichou |
|  |  | Kawaichou |
|  | Chuwa | Yamatotakada-shi |
|  |  | Kashihara-shi |
|  |  | Gose-shi |
|  |  | Kashiba-shi |
|  |  | Katsuragi-shi |
|  |  | Takatorichou |
|  |  | Asukamura |
|  |  | Koryochou |
|  | Nanwa | Gojo-shi |
|  |  | Yoshinochou |
|  |  | Oyodochou |
|  |  | Shimoichichou |
|  |  | Kurotakimura |
|  |  | Tenkawamura |
|  |  | Nosegawamura |
|  |  | Totsukawamura |
|  |  | Shimokitayamamura |
|  |  | Kamikitayamamura |
|  |  | Kawakamimura |
|  |  | Higashiyoshinomura |
| Wakayama | Wakayama | Wakayama-shi |
|  |  | Kainan-shi |
|  |  | Kiminochou |
|  | Naga | Kinokawa-shi |
|  |  | Iwade-shi |
|  | Hashimoto | Hashimoto-shi |
|  |  | Katsuragichou |
|  |  | Kudoyamachou |
|  |  | Koyachou |
|  | Arita | Arida-shi |
|  |  | Yuasachou |
|  |  | Hirokawachou |
|  |  | Arigawachou |
|  | Gobou | Gobo-shi |
|  |  | Mihamachou |
|  |  | Hidakachou |
|  |  | Yurachou |
|  |  | Inamichou |
|  |  | Hidakagawachou |
|  | Tanabe | Tanabe-shi |
|  |  | Minabechou |
|  |  | Shirahamachou |
|  |  | Kamitondachou |
|  |  | Susamichou |
|  | Shinguu | Shingu-shi |
|  |  | Nachikatsuurachou |
|  |  | Taijichou |
|  |  | Kozagawachou |
|  |  | Kitayamamura |
|  |  | Kushimotochou |
| Tottori | Toubu | Tottori-shi |
|  |  | Iwamichou |
|  |  | Wakasachou |
|  |  | Chizuchou |
|  |  | Yazuchou |
|  | Chubu | Kurayoshi-shi |
|  |  | Misasachou |
|  |  | Yurihamachou |
|  |  | Kotourachou |
|  |  | Hokueichou |
|  | Seibu | Yonago-shi |
|  |  | Sakaiminato-shi |
|  |  | Hiezuson |
|  |  | Daisenchou |
|  |  | Nanbuchou |
|  |  | Houkichou |
|  |  | Nichinanchou |
|  |  | Hinochou |
|  |  | Kofuchou |
| Shimane | Matsue | Matsue-shi |
|  |  | Yasugi-shi |
|  | Unnan | Unnan-shi |
|  |  | Okuizumochou |
|  |  | Iinanchou |
|  | Izumo | Izumo-shi |
|  | Ota | Oda-shi |
|  |  | Kawamotomachi |
|  |  | Misatochou |
|  |  | Ohnanchou |
|  | Hamada | Hamada-shi |
|  |  | Gotsu-shi |
|  | Masuda | Masuda-shi |
|  |  | Tsuwanochou |
|  |  | Yoshikachou |
|  | Oki | Amachou |
|  |  | Nishinoshimachou |
|  |  | Chibumura |
|  |  | Okinoshimachou |
| Okayama | Kennan-toubu | Okayama-shi Kita-ku |
|  |  | Okayama-shi Naka-ku |
|  |  | Okayama-shi Higashi-ku |
|  |  | Okayama-shi Minami-ku |
|  |  | Tamano-shi |
|  |  | Bizen-shi |
|  |  | Setochi-shi |
|  |  | Akaiwa-shi |
|  |  | Wakechou |
|  |  | Kibichuochou |
|  | Kennan-seibu | Kurashiki-shi |
|  |  | Kasaoka-shi |
|  |  | Ibara-shi |
|  |  | Soja-shi |
|  |  | Asakuchi-shi |
|  |  | Hayashimachou |
|  |  | Satoshochou |
|  |  | Yakagechou |
|  | Takahashi, Niimi | Takahashi-shi |
|  |  | Niimi-shi |
|  | Maniwa | Maniwa-shi |
|  |  | Shinjoson |
|  | Tsuyama, Aida | Tsuyama-shi |
|  |  | Mimasaka-shi |
|  |  | Kagaminochou |
|  |  | Shoochou |
|  |  | Nagichou |
|  |  | Nishiawakurason |
|  |  | Kumenanchou |
|  |  | Misakichou |
| Hiroshima | Hiroshima | Hiroshima-shi Naka-ku |
|  |  | Hiroshima-shi Higashi-ku |
|  |  | Hiroshima-shi Minami-ku |
|  |  | Hiroshima-shi Nishi-ku |
|  |  | Hiroshima-shi Asaminami-ku |
|  |  | Hiroshima-shi Asakita-ku |
|  |  | Hiroshima-shi Aki-ku |
|  |  | Hiroshima-shi Saeki-ku |
|  |  | Akitakata-shi |
|  |  | Fuchuchou |
|  |  | Kaitachou |
|  |  | Kumanochou |
|  |  | Sakachou |
|  |  | Akiotachou |
|  |  | Kitahiroshimachou |
|  | Hiroshima-nishi | Otake-shi |
|  |  | Hatsukaichi-shi |
|  | Kure | Kure-shi |
|  |  | Etajima-shi |
|  | Hiroshima-chuou | Takehara-shi |
|  |  | Higashihiroshima-shi |
|  |  | Osakikamijimachou |
|  | Bisan | Mihara-shi |
|  |  | Onomichi-shi |
|  |  | Serachou |
|  | Fukuyama, fuchu | Fukuyama-shi |
|  |  | Fuchu-shi |
|  |  | Jinsekikogenchou |
|  | Bihoku | Miyoshi-shi |
|  |  | Shobara-shi |
| Yamaguchi | Iwakuni | Iwakuni-shi |
|  |  | Wakichou |
|  | Yanai | Yanai-shi |
|  |  | Suo-Oshimachou |
|  |  | Kaminosekichou |
|  |  | Tabusechou |
|  |  | Hiraochou |
|  | Shunan | Kudamatsu-shi |
|  |  | Hikari-shi |
|  |  | Shunan-shi |
|  | Yamaguchi, Houhu | Yamaguchi-shi |
|  |  | Hofu-shi |
|  | Ube, Onoda | Ube-shi |
|  |  | Mine-shi |
|  |  | Sanyoonoda-shi |
|  | Shimonoseki | Shimonoseki-shi |
|  | Nagato | Nagato-shi |
|  | Hagi | Hagi-shi |
|  |  | Abuchou |
| Tokushima | Toubu | Tokushima-shi |
|  |  | Naruto-shi |
|  |  | Yoshinogawa-shi |
|  |  | Awa-shi |
|  |  | Sanagouchison |
|  |  | Ishiichou |
|  |  | Kamiyamachou |
|  |  | Matsushigechou |
|  |  | Kitajimachou |
|  |  | Aizumichou |
|  |  | Itanochou |
|  |  | Kamikitachou |
|  | Nanbu | Komatsushima-shi |
|  |  | Anan-shi |
|  |  | Katsuurachou |
|  |  | Kamikatsuchou |
|  |  | Nakachou |
|  |  | Mugichou |
|  |  | Minamichou |
|  |  | Kaiyochou |
|  | Seibu | Mima-shi |
|  |  | Miyoshi-shi |
|  |  | Tsurugichou |
|  |  | Higashimiyoshichou |
| Kagawa | Okawa | Sanuki-shi |
|  |  | Higashikagawa-shi |
|  | Syozu | Tonoshochou |
|  |  | Shodoshimachou |
|  | Takamatsu | Takamatsu-shi |
|  |  | Mikichou |
|  |  | Naoshimachou |
|  | Chusan | Marugame-shi |
|  |  | Sakaide-shi |
|  |  | Zentsuji-shi |
|  |  | Utazuchou |
|  |  | Ayagawachou |
|  |  | Kotohirachou |
|  |  | Tadotsuchou |
|  |  | Mannochou |
|  | Mitoyo | Kanonji-shi |
|  |  | Mitoyo-shi |
| Ehime | Uma | Shikokuchuo-shi |
|  | Nihama, Saijo | Niihama-shi |
|  |  | Saijo-shi |
|  | Imabari | Imabari-shi |
|  |  | Kamijimachou |
|  | Matsuyama | Matsuyama-shi |
|  |  | Iyo-shi |
|  |  | Toon-shi |
|  |  | Kumakogenchou |
|  |  | Masakichou |
|  |  | Tobechou |
|  | Yawatahama, Ozu | Yawatahama-shi |
|  |  | Ozu-shi |
|  |  | Seiyo-shi |
|  |  | Ushikochou |
|  |  | Ikatachou |
|  | Uwajima | Uwajima-shi |
|  |  | Matsunochou |
|  |  | Kihokuchou |
|  |  | Ainanchou |
| Kochi | Aki | Muroto-shi |
|  |  | Aki-shi |
|  |  | Toyochou |
|  |  | Naharichou |
|  |  | Tanochou |
|  |  | Yasudachou |
|  |  | Kitagawamura |
|  |  | Umajimura |
|  |  | Geiseimura |
|  | Chuou | Kochi-shi |
|  |  | Nankoku-shi |
|  |  | Tosa-shi |
|  |  | Konan-shi |
|  |  | Kami-shi |
|  |  | Motoyamachou |
|  |  | Otoyochou |
|  |  | Tosachou |
|  |  | Okawamura |
|  |  | Inochou |
|  |  | Niyodogawachou |
|  |  | Sagawachou |
|  |  | Ochichou |
|  |  | Hidakamura |
|  | Takahata | Susaki-shi |
|  |  | Nakatosachou |
|  |  | Yusuharachou |
|  |  | Tsunochou |
|  |  | Shimantochou |
|  | Hata | Sukumo-shi |
|  |  | Tosashimizu-shi |
|  |  | Shimanto-shi |
|  |  | Otsukichou |
|  |  | Miharamura |
|  |  | Kuroshiochou |
| Fukuoka | Fukuoka, Itoshima | Fukuoka-shi Higashi-ku |
|  |  | Fukuoka-shi Hakata-ku |
|  |  | Fukuoka-shi Chuou-ku |
|  |  | Fukuoka-shi Minami-ku |
|  |  | Fukuoka-shi Nishi-ku |
|  |  | Fukuoka-shi Jonan-ku |
|  |  | Fukuoka-shi Sawara-ku |
|  |  | Itoshima-shi |
|  | Kasuya | Koga-shi |
|  |  | Umimachi |
|  |  | Sasagurimachi |
|  |  | Shimemachi |
|  |  | Suemachi |
|  |  | Shingumachi |
|  |  | Hisayamamachi |
|  |  | Kasuyamachi |
|  | Munakata | Munakata-shi |
|  |  | Fukutsu-shi |
|  | Tsukushi | Chikushino-shi |
|  |  | Kasuga-shi |
|  |  | Onojo-shi |
|  |  | Dazaifu-shi |
|  |  | Nakagawamachi |
|  | Asakura | Asakura-shi |
|  |  | Chikuzenmachi |
|  |  | Tohomura |
|  | Kurume | Kurume-shi |
|  |  | Okawa-shi |
|  |  | Ogori-shi |
|  |  | Ukiha-shi |
|  |  | Tachiaraimachi |
|  |  | Ookimachi |
|  | Yame, Chikugo | Yame-shi |
|  |  | Chikugo-shi |
|  |  | Hirokawamachi |
|  | Ariake | Omuta-shi |
|  |  | Yanagawa-shi |
|  |  | Miyama-shi |
|  | Iizuka | Iizuka-shi |
|  |  | Kama-shi |
|  |  | Keisenmachi |
|  | Noogata, Kurate | Nogata-shi |
|  |  | Miyawaka-shi |
|  |  | Kotakemachi |
|  |  | Kuratemachi |
|  | Tagawa | Tagawa-shi |
|  |  | Kawaramachi |
|  |  | Soedamachi |
|  |  | Itodamachi |
|  |  | Kawasakimachi |
|  |  | Otomachi |
|  |  | Akamuramachi |
|  |  | Fukumachi |
|  | Kitakyushu | Kitakyushu-shi Moji-ku |
|  |  | Kitakyushu-shi Wakamatsu-ku |
|  |  | Kitakyushu-shi Tobata-ku |
|  |  | Kitakyushu-shi Kokurakita-ku |
|  |  | Kitakyushu-shi Kokuraminami-ku |
|  |  | Kitakyushu-shi Yahatahigashi-ku |
|  |  | Kitakyushu-shi Yahatanishi-ku |
|  |  | Nakama-shi |
|  |  | Ashiyama-chi |
|  |  | Mizumakimachi |
|  |  | Okagakimachi |
|  |  | Ongachou |
|  | Keichiku | Yukuhashi-shi |
|  |  | Buzen-shi |
|  |  | Kandamachi |
|  |  | Miyakomachi |
|  |  | Yoshitomimachi |
|  |  | Kogemachi |
|  |  | Chikujomachi |
| Saga | Chubu | Saga-shi |
|  |  | Taku-shi |
|  |  | Ogi-shi |
|  |  | Kanzaki-shi |
|  |  | Yoshinogarichou |
|  | Toubu | Tosu-shi |
|  |  | Kiyamachou |
|  |  | Kamiminechou |
|  |  | Miyakichou |
|  | Hokubu | Karatsu-shi |
|  |  | Genkaichou |
|  | Seibu | Imari-shi |
|  |  | Aritachou |
|  | Nanbu | Takeo-shi |
|  |  | Kashima-shi |
|  |  | Ureshino-shi |
|  |  | Omachichou |
|  |  | Kouhokumachi |
|  |  | Shiroishichou |
|  |  | Tarachou |
| Nagasaki | Ngasaki | Nagasaki-shi |
|  |  | Saikai-shi |
|  |  | Nagayochou |
|  |  | Togitsuchou |
|  | Sasebo-kenhoku | Sasebo-shi |
|  |  | Hirado-shi |
|  |  | Matsura-shi |
|  |  | Sazachou |
|  | Kennou | Isahaya-shi |
|  |  | Omura-shi |
|  |  | Higashisonogichou |
|  |  | Kawatanachou |
|  |  | Hasamichou |
|  | Kennan | Shimabara-shi |
|  |  | Unzen-shi |
|  |  | Minamishimabara-shi |
|  | Gotou | Goto-shi |
|  | Kamigotou | Ojikachou |
|  |  | Shinkamigotochou |
|  | Iki | Iki-shi |
|  | Tsushima | Tsushima-shi |
| Kumamoto | Kumamoto | Kumamotochi Chuou-ku |
|  |  | Kumamotochi Higashi-ku |
|  |  | Kumamotochi Nishi-ku |
|  |  | Kumamotochi Minami-ku |
|  |  | Kumamotochi Kita-ku |
|  | Uki | Uto-shi |
|  |  | Uki-shi |
|  |  | Misatomachi |
|  | Ariake | Arao-shi |
|  |  | Tamana-shi |
|  |  | Gyokutomachi |
|  |  | Nankanmachi |
|  |  | Nagasumachi |
|  |  | Nagomimachi |
|  | Kamoto | Yamaga-shi |
|  | Kikuchi | Kikuchi-shi |
|  |  | Koshi-shi |
|  |  | Ozumachi |
|  |  | Kikuyomachi |
|  | Aso | Aso-shi |
|  |  | Minamiogunimachi |
|  |  | Ogunimachi |
|  |  | Ubuyamamachi |
|  |  | Takamorimachi |
|  |  | Nishiharamachi |
|  |  | Minamiasomura |
|  | Kamimashiki | Mifunemachi |
|  |  | Kashimamachi |
|  |  | Mashikimachi |
|  |  | Kosamachi |
|  |  | Yamatochou |
|  | Yatsushiro | Yatsushiro-shi |
|  |  | Hikawachou |
|  | Ashikita | Minamata-shi |
|  |  | Ashikitamachi |
|  |  | Tsunagimachi |
|  | Kuma | Hitoyoshi-shi |
|  |  | Nishikimachi |
|  |  | Taragimachi |
|  |  | Yunomaemachi |
|  |  | Mizukamimura |
|  |  | Sagaramura |
|  |  | Itsukimura |
|  |  | Yamaemura |
|  |  | Kumamura |
|  |  | Asagirichou |
|  | Amakusa | Kamiamakusa-shi |
|  |  | Amakusa-shi |
|  |  | Reihokumachi |
| Oita | Toubu | Beppu-shi |
|  |  | Kitsuki-shi |
|  |  | Kunisak-ishi |
|  |  | Himeshimamura |
|  |  | Hijimachi |
|  | Chubu | Oita-shi |
|  |  | Usuki-shi |
|  |  | Tsukumi-shi |
|  |  | Yufu-shi |
|  | Nanbu | Saiki-shi |
|  | Houhi | Taketa-shi |
|  |  | Bungoono-shi |
|  | Seibu | Hita-shi |
|  |  | Kokonoemachi |
|  |  | Kusumachi |
|  | Hokubu | Nakatsu-shi |
|  |  | Bungotakada-shi |
|  |  | Usa-shi |
| Miyazaki | Miyazaki-higashimorokata | Miyazaki-shi |
|  |  | Kunitomichou |
|  |  | Ayachou |
|  | Miyakonojou-kitamorokata | Miyakonojo-shi |
|  |  | Mimatachou |
|  | Nobeoka-nishiusuki | Nobeoka-shi |
|  |  | Takachihochou |
|  |  | Hinokagechou |
|  |  | Gokasechou |
|  | Nichinankushima | Nichinan-shi |
|  |  | Kushima-shi |
|  | Nishimoro | Kobayashi-shi |
|  |  | Ebino-shi |
|  |  | Takaharuchou |
|  | Saitokoyu | Saito-shi |
|  |  | Takanabechou |
|  |  | Shintomichou |
|  |  | Nishimerason |
|  |  | Kijochou |
|  |  | Kawaminamichou |
|  |  | Tsunochou |
|  | Hyugairigou | Hyuga-shi |
|  |  | Kadokawachou |
|  |  | Morotsukason |
|  |  | Shibason |
|  |  | Misatochou |
| Kagoshima | Kagoshima | Kagoshima-shi |
|  |  | Hioki-shi |
|  |  | Ichikikushikino-shi |
|  |  | Mishimamura |
|  |  | Toshimamura |
|  | Nansatsu | Makurazaki-shi |
|  |  | Ibusuki-shi |
|  |  | Minamisatsuma-shi |
|  |  | Minamikyushu-shi |
|  | Sensatsu | Satsumasendai-shi |
|  |  | Satsumachou |
|  | Izumi | Akune-shi |
|  |  | Izumi-shi |
|  |  | Nagashimachou |
|  | Aira, Isa | Kirishima-shi |
|  |  | Isa-shi |
|  |  | Aira-shi |
|  |  | Yusuichou |
|  | Soo | So-shi |
|  |  | Shibushi-shi |
|  |  | Osakichou |
|  | Kimotsuki | Kanoya-shi |
|  |  | Tarumizu-shi |
|  |  | Higashikushirachou |
|  |  | Kinkochou |
|  |  | Minamiosumichou |
|  |  | Kimotsukichou |
|  | Kumage | Nishinomote-shi |
|  |  | Nakatanechou |
|  |  | Minamitanechou |
|  |  | Yakushimachou |
|  | Amami | Amami-shi |
|  |  | Yamatoson |
|  |  | Ukenson |
|  |  | Setouchichou |
|  |  | Tatsugochou |
|  |  | Kikaichou |
|  |  | Tokunoshimachou |
|  |  | Amagichou |
|  |  | Isenchou |
|  |  | Wadomatichou |
|  |  | Chinachou |
|  |  | Yoronchou |
| Okinawa | Hokubu | Nago-shi |
|  |  | Kunigamison |
|  |  | Ogimison |
|  |  | Higashison |
|  |  | Nakijinson |
|  |  | Motobuchou |
|  |  | Ieson |
|  |  | Iheyason |
|  |  | Izenason |
|  | Chubu | Ginowan-shi |
|  |  | Okinawa-shi |
|  |  | Uruma-shi |
|  |  | Onnason |
|  |  | Ginozason |
|  |  | Kinchou |
|  |  | Yomitanson |
|  |  | Kadenachou |
|  |  | Chatanchou |
|  |  | Kitanakagusukuson |
|  |  | Nakagusukuson |
|  | Nanbu | Naha-shi |
|  |  | Urasoe-shi |
|  |  | Itoman-shi |
|  |  | Tomigusuku-shi |
|  |  | Nanjo-shi |
|  |  | Nishiharachou |
|  |  | Yonabaruchou |
|  |  | Haebaruchou |
|  |  | Tokashikison |
|  |  | Zamamison |
|  |  | Agunison |
|  |  | Tonakison |
|  |  | Minamidaitoson |
|  |  | Kitadaitoson |
|  |  | Kumejimachou |
|  |  | Yaesechou |
|  | Miyako | Miyakojima-shi |
|  |  | Taramason |
|  | Yaeyama | Ishigaki-shi |
|  |  | Taketomichou |
|  |  | Yonagunichou |
