# Supplementary material for: Effect of population inflow and outflow between rural and urban areas on regional antimicrobial use surveillance
Source: PLoS One. 2021 Mar 18;16(3):e0248338. doi: 10.1371/journal.pone.0248338 (PMC7971456; doi:10.1371/journal.pone.0248338)
Supplement: S2 Table — (DOCX) [file pone.0248338.s002.docx]

**S2 Table. Daytime and nighttime populations at the national level, prefectural level, and secondary medical area level in Japan**

| Area | Daytime population | Nighttime population |
| --- | --- | --- |
| National | 127,094,745 | 127,094,745 |
| Prefectures | | |
| Hokkaido | 5,378,786 | 5,381,733 |
| Aomori | 1,306,283 | 1,308,265 |
| Iwate | 1,276,705 | 1,279,594 |
| Miyagi | 2,339,786 | 2,333,899 |
| Akita | 1,020,642 | 1,023,119 |
| Yamagata | 1,120,365 | 1,123,891 |
| Fukushima | 1,917,815 | 1,914,039 |
| Ibaraki | 2,842,851 | 2,916,976 |
| Tochigi | 1,955,198 | 1,974,255 |
| Gunma | 1,969,764 | 1,973,115 |
| Saitama | 6,456,452 | 7,266,534 |
| Chiba | 5,582,241 | 6,222,666 |
| Tokyo | 15,920,405 | 13,515,271 |
| Kanagawa | 8,322,926 | 9,126,214 |
| Niigata | 2,302,236 | 2,304,264 |
| Toyama | 1,063,834 | 1,066,328 |
| Ishikawa | 1,156,536 | 1,154,008 |
| Fukui | 786,736 | 786,740 |
| Yamanashi | 828,495 | 834,930 |
| Nagano | 2,094,051 | 2,098,804 |
| Gifu | 1,952,630 | 2,031,903 |
| Shizuoka | 3,692,336 | 3,700,305 |
| Aichi | 7,586,294 | 7,483,128 |
| Mie | 1,784,775 | 1,815,865 |
| Shiga | 1,363,716 | 1,412,916 |
| Kyoto | 2,656,353 | 2,610,353 |
| Osaka | 9,224,306 | 8,839,469 |
| Hyogo | 5,294,074 | 5,534,800 |
| Nara | 1,228,426 | 1,364,316 |
| Wakayama | 946,387 | 963,579 |
| Tottori | 572,678 | 573,441 |
| Shimane | 694,770 | 694,352 |
| Okayama | 1,921,822 | 1,921,525 |
| Hiroshima | 2,850,087 | 2,843,990 |
| Yamaguchi | 1,399,109 | 1,404,729 |
| Tokushima | 752,919 | 755,733 |
| Kagawa | 978,511 | 976,263 |
| Ehime | 1,385,392 | 1,385,262 |
| Kochi | 727,644 | 728,276 |
| Fukuoka | 5,105,438 | 5,101,556 |
| Saga | 834,871 | 832,832 |
| Nagasaki | 1,374,210 | 1,377,187 |
| Kumamoto | 1,777,292 | 1,786,170 |
| Oita | 1,165,668 | 1,166,338 |
| Miyazaki | 1,103,434 | 1,104,069 |
| Kagoshima | 1,646,386 | 1,648,177 |
| Okinawa | 1,433,110 | 1,433,566 |
| Secondary medical areas | | |
| Minamioshima | 380,381 | 381,620 |
| Minamihiyama | 23,616 | 23,769 |
| Kitaoshimahiyama | 37,321 | 37,279 |
| Sapporo | 2,372,060 | 2,375,449 |
| Shiribeshi | 218,249 | 215,522 |
| Minamisorachi | 163,350 | 166,691 |
| Nakasorachi | 108,275 | 108,970 |
| Kitasorachi | 32,871 | 32,675 |
| Nishiiburi | 190,249 | 189,696 |
| Higashiiburi | 213,848 | 212,059 |
| Hidaka | 68,906 | 69,015 |
| Kamikawa-chubu | 392,927 | 394,270 |
| Kamikawa-hokubu | 66,841 | 66,591 |
| Hurano | 43,194 | 42,597 |
| Rumoi | 48,011 | 47,912 |
| Souya | 67,783 | 67,503 |
| Hokumou | 222,860 | 222,696 |
| Enmon | 71,094 | 70,846 |
| Tokachi | 343,394 | 343,436 |
| Kushiro | 236,585 | 236,516 |
| Nemuro | 76,971 | 76,621 |
| Tsugaru-Chiiki | 291,538 | 291,789 |
| Hachinohe-Chiiki | 321,979 | 323,447 |
| Aomori-Chiiki | 313,335 | 310,640 |
| Seihokugo-Chiiki | 127,412 | 131,631 |
| Kamitousan-Chiiki | 178,678 | 176,307 |
| Shimokita-Chiiki | 73,341 | 74,451 |
| Morioka | 477,499 | 476,758 |
| Iwate-chubu | 224,031 | 225,155 |
| Tankou | 133,855 | 135,317 |
| Ryoban | 128,344 | 129,451 |
| Kesen | 63,749 | 63,536 |
| Kamaichi | 50,580 | 48,561 |
| Miyako | 86,241 | 85,809 |
| Kuji | 56,804 | 59,279 |
| Ninohe | 55,602 | 55,728 |
| Sennan | 169,680 | 177,192 |
| Sendai | 1,555,720 | 1,528,508 |
| Osaki, Kurihara | 264,052 | 275,831 |
| Ishinomaki, Tomeshi, Kesennuma | 350,334 | 352,368 |
| Odate, Kaduno | 112,209 | 111,552 |
| Kitaakita | 34,967 | 35,605 |
| Noshiro, Yamamoto | 81,462 | 82,476 |
| Akita-Shuhen | 405,636 | 400,911 |
| Yurihonjou, Nikaho | 103,346 | 105,251 |
| Daisen, Senboku | 126,424 | 130,585 |
| Yokote | 93,863 | 92,197 |
| Yuzawa, Ogatsu | 62,735 | 64,542 |
| Murayama | 551,674 | 551,524 |
| Mogami | 76,170 | 77,895 |
| Okitama | 213,329 | 214,975 |
| Shonai | 279,192 | 279,497 |
| Kenhoku | 486,268 | 490,647 |
| Kennaka | 539,131 | 539,376 |
| Kennan | 144,228 | 144,080 |
| Aizu | 248,367 | 250,605 |
| Minamiaizu | 26,647 | 27,149 |
| Soso | 128,789 | 111,945 |
| Iwaki | 344,385 | 350,237 |
| Mito | 482,175 | 468,040 |
| Hitachi | 267,075 | 259,104 |
| Hitachioota, Hitachinaka | 335,587 | 360,612 |
| Rokkou | 273,354 | 274,568 |
| Tsuchiura | 260,434 | 258,971 |
| Tsukuba | 350,710 | 337,582 |
| Toride, Ryugasaki | 405,990 | 465,650 |
| Chikusei, Simotsuma | 248,815 | 264,113 |
| Koga, Bandou | 218,711 | 228,336 |
| Kenhoku | 366,110 | 380,922 |
| Kensei | 175,565 | 181,760 |
| Utsunomiya | 537,708 | 518,594 |
| Kentou | 149,873 | 142,917 |
| Kennan | 462,564 | 481,691 |
| Ryomou | 263,378 | 268,371 |
| Maebashi | 351,420 | 336,154 |
| Shibukawa | 103,087 | 113,800 |
| Isezaki | 237,177 | 245,468 |
| Takasaki, Annaka | 431,960 | 429,415 |
| Fujioka | 65,340 | 68,892 |
| Tomioka | 70,522 | 72,489 |
| Agatuma | 55,601 | 56,391 |
| Numata | 80,807 | 83,407 |
| Kiryu | 157,435 | 165,620 |
| Ota, Tatebayashi | 416,415 | 401,479 |
| Nanbu | 658,230 | 786,522 |
| Nanseibu | 596,025 | 709,451 |
| Toubu | 978,490 | 1,140,278 |
| Saitama | 1,175,579 | 1,263,979 |
| Kenou | 440,871 | 529,055 |
| Kawagoehiki | 753,453 | 800,002 |
| Seibu | 692,089 | 778,416 |
| Tone | 573,736 | 647,166 |
| Hokubu | 492,988 | 510,017 |
| Chichibu | 94,991 | 101,648 |
| Chiba | 951,528 | 971,882 |
| Toukatsu-nanbu | 1,483,292 | 1,738,624 |
| Toukatsu-hokubu | 1,149,426 | 1,356,996 |
| Inba | 641,951 | 710,071 |
| Katorikaisou | 262,612 | 280,770 |
| Sanmushichouseiisumi | 395,123 | 434,489 |
| Awa | 125,815 | 128,451 |
| Kimitsu | 314,638 | 326,727 |
| Ichihara | 257,856 | 274,656 |
| Ku-chuoubu | 3,052,519 | 860,669 |
| Ku-nanbu | 1,237,887 | 1,103,937 |
| Ku-seinanbu | 1,689,811 | 1,405,501 |
| Ku-seibu | 1,568,794 | 1,225,772 |
| Ku-seihokubu | 1,860,082 | 1,915,881 |
| Ku-touhokubu | 1,175,307 | 1,325,299 |
| Ku-toubu | 1,449,192 | 1,435,681 |
| Nishitama | 359,764 | 390,897 |
| Minamitama | 1,353,103 | 1,430,411 |
| Kitatama-seibu | 615,635 | 640,617 |
| Kitatama-nanbu | 930,058 | 1,022,646 |
| Kitatama-hokubu | 600,994 | 731,469 |
| Tousyo | 27,259 | 26,491 |
| Yokohama-hokubu | 1,400,713 | 1,570,303 |
| Yokohama-seibu | 1,020,225 | 1,105,037 |
| Yokohama-nanbu | 995,122 | 1,049,504 |
| Kawasaki-hokubu | 666,015 | 843,416 |
| Kawasaki-nanbu | 636,472 | 631,797 |
| Yokosuka, Miura | 646,934 | 714,415 |
| Shonan-toubu | 629,392 | 711,178 |
| Shonan-seibu | 543,201 | 587,047 |
| Kenou | 813,368 | 845,580 |
| Sagamihara | 636,218 | 720,780 |
| Kensei | 335,266 | 347,157 |
| Kaetsu | 209,421 | 211,493 |
| Niigata | 918,699 | 916,656 |
| Kenou | 225,972 | 227,225 |
| Chuetsu | 450,970 | 448,375 |
| Uonuma | 167,062 | 168,912 |
| Jouetsu | 272,689 | 274,348 |
| Sado | 57,423 | 57,255 |
| Nikawa | 119,600 | 121,507 |
| Toyama | 516,037 | 501,670 |
| Takaoka | 301,370 | 312,425 |
| Tonami | 126,827 | 130,726 |
| Minamikaga | 224,888 | 229,333 |
| Ishikawa-chuou | 738,758 | 728,259 |
| Noto-chubu | 125,115 | 128,221 |
| Noto-hokubu | 67,775 | 68,195 |
| Fukui, Sakai | 417,890 | 404,796 |
| Okuetsu | 52,214 | 57,234 |
| Tannan | 175,300 | 184,783 |
| Reinan | 141,332 | 139,927 |
| Chuhoku | 477,490 | 464,759 |
| Kyoutou | 125,344 | 136,371 |
| Kyonan | 49,140 | 52,771 |
| Fuji, Toubu | 176,521 | 181,029 |
| Saku | 206,847 | 209,016 |
| Kamiko | 198,047 | 197,443 |
| Suwa | 198,846 | 198,475 |
| Kamiina | 181,163 | 184,305 |
| Hanni | 161,839 | 162,200 |
| Kiso | 28,209 | 28,399 |
| Matsumoto | 432,029 | 427,928 |
| Taihoku | 56,632 | 59,748 |
| Nagano | 545,730 | 543,424 |
| Hokushin | 84,709 | 87,866 |
| Gifu | 773,918 | 799,766 |
| Seinou | 352,843 | 372,399 |
| Chunou | 361,300 | 373,712 |
| Tounou | 315,425 | 336,954 |
| Hida | 149,144 | 149,072 |
| Kamo | 65,467 | 66,438 |
| Atamiitou | 105,785 | 105,889 |
| Suntoutagata | 656,268 | 657,570 |
| Fuji | 371,292 | 379,169 |
| Shizuoka | 726,136 | 704,989 |
| Shidahaibara | 441,307 | 463,011 |
| Chutouen | 466,759 | 465,470 |
| Seibu | 859,322 | 857,769 |
| Nagoya | 2,589,799 | 2,295,638 |
| Ama | 290,271 | 329,158 |
| Owari-chubu | 159,169 | 166,637 |
| Owari-toubu | 426,567 | 467,393 |
| Owari-seibu | 460,314 | 517,735 |
| Owari-hokubu | 708,173 | 733,279 |
| Chitahantou | 582,081 | 620,905 |
| Nishimikawa-hokubu | 530,702 | 484,352 |
| Nishimikawa-nanbunishi | 709,948 | 689,978 |
| Nishimikawa-nanbuhigashi | 393,900 | 420,600 |
| Higashimikawa-hokubu | 55,392 | 56,788 |
| Higashimikawa-nanbu | 679,978 | 700,665 |
| Hokusei | 824,139 | 841,029 |
| Chusei-iga | 453,043 | 449,262 |
| Nansei-shima | 437,523 | 453,957 |
| Higashikishuu | 70,070 | 71,617 |
| Otsu | 310,543 | 340,973 |
| Konan | 334,383 | 333,744 |
| Kouga | 143,126 | 145,190 |
| Higashioumi | 219,610 | 229,799 |
| Kotou | 157,374 | 156,273 |
| Kohoku | 151,379 | 156,912 |
| Kosai | 47,301 | 50,025 |
| Tango | 94,881 | 97,424 |
| Chutan | 201,021 | 196,746 |
| Nantan | 124,730 | 137,077 |
| Kyoto, Otokuni | 1,737,593 | 1,623,834 |
| Yamashiro-kita | 405,577 | 438,080 |
| Yamashiro-minami | 92,551 | 117,192 |
| Toyono | 945,650 | 1,036,617 |
| Mishima | 684,135 | 746,852 |
| Kitakawachi | 1,068,985 | 1,164,015 |
| Nakakawachi | 843,278 | 842,696 |
| Minamikawachi | 535,002 | 612,886 |
| Sakai-shi | 785,324 | 839,310 |
| Senshu | 818,483 | 905,908 |
| Osaka-shi | 3,543,449 | 2,691,185 |
| Kobe | 1,571,625 | 1,537,272 |
| Hanshin-minami | 954,144 | 1,035,763 |
| Hanshin-kita | 610,106 | 721,690 |
| Higashiharima | 650,569 | 716,633 |
| Kitaharima | 274,684 | 272,447 |
| Nakaharima | 581,225 | 579,154 |
| Nishiharima | 246,227 | 260,312 |
| Tajima | 169,844 | 170,232 |
| Tanba | 101,698 | 106,150 |
| Awaji | 133,952 | 135,147 |
| Nara | 341,656 | 360,310 |
| Touwa | 192,867 | 209,741 |
| Seiwa | 295,140 | 345,503 |
| Chuwa | 327,601 | 376,197 |
| Nanwa | 71,162 | 72,565 |
| Wakayama | 438,625 | 425,220 |
| Naga | 99,349 | 116,068 |
| Hashimoto | 78,173 | 88,342 |
| Arita | 70,562 | 74,255 |
| Gobou | 62,149 | 63,603 |
| Tanabe | 128,354 | 128,161 |
| Shinguu | 69,175 | 67,930 |
| Toubu | 233,052 | 232,610 |
| Chubu | 103,266 | 104,320 |
| Seibu | 236,360 | 236,511 |
| Matsue | 251,980 | 245,758 |
| Unnan | 54,245 | 57,126 |
| Izumo | 168,930 | 171,938 |
| Ota | 53,899 | 54,609 |
| Hamada | 83,337 | 82,573 |
| Masuda | 61,752 | 61,745 |
| Oki | 20,627 | 20,603 |
| Kennan-toubu | 940,686 | 921,940 |
| Kennan-seibu | 688,361 | 707,450 |
| Takahashi, Niimi | 64,277 | 62,733 |
| Maniwa | 45,858 | 46,990 |
| Tsuyama, Aida | 182,640 | 182,412 |
| Hiroshima | 1,384,674 | 1,365,134 |
| Hiroshima-nishi | 132,629 | 142,771 |
| Kure | 248,928 | 252,891 |
| Hiroshima-chuou | 225,796 | 227,325 |
| Bisan | 250,994 | 251,157 |
| Fukuyama, fuchu | 515,203 | 514,097 |
| Bihoku | 91,863 | 90,615 |
| Iwakuni | 141,396 | 143,042 |
| Yanai | 79,409 | 81,062 |
| Shunan | 254,755 | 252,023 |
| Yamaguchi, Houhu | 314,634 | 313,364 |
| Ube, Onoda | 257,019 | 258,259 |
| Shimonoseki | 264,983 | 268,517 |
| Nagato | 34,753 | 35,439 |
| Hagi | 52,160 | 53,023 |
| Toubu | 528,153 | 527,175 |
| Nanbu | 145,375 | 147,656 |
| Seibu | 79,391 | 80,902 |
| Okawa | 77,803 | 81,303 |
| Syozu | 28,710 | 28,864 |
| Takamatsu | 464,354 | 451,571 |
| Chusan | 286,834 | 289,592 |
| Mitoyo | 120,810 | 124,933 |
| Uma | 88,166 | 87,413 |
| Nihama, Saijo | 226,917 | 228,077 |
| Imabari | 167,059 | 165,249 |
| Matsuyama | 645,777 | 646,055 |
| Yawatahama, Ozu | 143,942 | 144,324 |
| Uwajima | 113,531 | 114,144 |
| Aki | 48,388 | 48,350 |
| Chuou | 536,648 | 536,869 |
| Takahata | 56,057 | 56,173 |
| Hata | 86,551 | 86,884 |
| Fukuoka, Itoshima | 1,782,524 | 1,635,156 |
| Kasuya | 258,660 | 283,544 |
| Munakata | 129,802 | 155,297 |
| Tsukushi | 367,501 | 433,521 |
| Asakura | 81,680 | 83,924 |
| Kurume | 438,210 | 456,196 |
| Yame, Chikugo | 132,111 | 132,930 |
| Ariake | 218,745 | 223,276 |
| Iizuka | 177,202 | 181,385 |
| Noogata, Kurate | 118,503 | 109,075 |
| Tagawa | 120,093 | 126,104 |
| Kitakyushu | 1,098,947 | 1,096,744 |
| Keichiku | 181,460 | 184,404 |
| Chubu | 354,967 | 348,633 |
| Toubu | 129,545 | 124,964 |
| Hokubu | 125,433 | 128,687 |
| Seibu | 77,047 | 75,386 |
| Nanbu | 147,879 | 155,162 |
| Ngasaki | 534,841 | 530,551 |
| Sasebo-kenhoku | 325,441 | 324,294 |
| Kennou | 263,386 | 268,091 |
| Kennan | 132,792 | 136,086 |
| Gotou | 37,247 | 37,327 |
| Kamigotou | 21,917 | 22,278 |
| Iki | 27,015 | 27,103 |
| Tsushima | 31,571 | 31,457 |
| Kumamoto | 756,852 | 740,822 |
| Uki | 100,456 | 107,115 |
| Ariake | 152,924 | 161,320 |
| Kamoto | 50,701 | 52,264 |
| Kikuchi | 179,148 | 180,973 |
| Aso | 64,660 | 64,393 |
| Kamimashiki | 83,594 | 85,768 |
| Yatsushiro | 137,401 | 139,466 |
| Ashikita | 47,111 | 47,745 |
| Kuma | 88,265 | 88,820 |
| Amakusa | 116,180 | 117,484 |
| Toubu | 205,454 | 211,019 |
| Chubu | 574,790 | 569,125 |
| Nanbu | 71,886 | 72,211 |
| Houhi | 58,699 | 58,916 |
| Seibu | 91,442 | 91,991 |
| Hokubu | 163,397 | 163,076 |
| Miyazaki-higashimorokata | 432,752 | 428,089 |
| Miyakonojou-kitamorokata | 191,797 | 190,433 |
| Nobeoka-nishiusuki | 146,953 | 145,747 |
| Nichinankushima | 71,739 | 72,869 |
| Nishimoro | 74,214 | 75,059 |
| Saitokoyu | 97,959 | 101,901 |
| Hyugairigou | 88,020 | 89,971 |
| Kagoshima | 685,169 | 679,508 |
| Nansatsu | 135,610 | 135,668 |
| Sensatsu | 120,085 | 118,476 |
| Izumi | 84,390 | 85,387 |
| Aira, Isa | 231,854 | 238,167 |
| Soo | 80,121 | 81,277 |
| Kimotsuki | 156,079 | 156,787 |
| Kumage | 42,851 | 42,760 |
| Amami | 110,227 | 110,147 |
| Hokubu | 103,999 | 101,444 |
| Chubu | 481,291 | 499,000 |
| Nanbu | 741,765 | 727,337 |
| Miyako | 52,433 | 52,380 |
| Yaeyama | 53,622 | 53,405 |
