# Supplementary material for: Effect of population inflow and outflow between rural and urban areas on regional antimicrobial use surveillance
Source: PLoS One. 2021 Mar 18;16(3):e0248338. doi: 10.1371/journal.pone.0248338 (PMC7971456; doi:10.1371/journal.pone.0248338)
Supplement: S3 Table — (DOCX) [file pone.0248338.s003.docx]

S3 Table. Defined Daily Doses of antimicrobials at the national level, prefectural level, and secondary medical area level in Japan

| **Area** | **DDDs** |
| --- | --- |
| National | 171232029.3 |
| Prefectures | |
| Hokkaido | 6627159.254 |
| Aomori | 1652595.063 |
| Iwate | 1384816.911 |
| Miyagi | 2826837.873 |
| Akita | 1310022.543 |
| Yamagata | 1352668.806 |
| Fukushima | 2392232.983 |
| Ibaraki | 3584395.086 |
| Tochigi | 2407488.286 |
| Gunma | 2374176.699 |
| Saitama | 8049651.204 |
| Chiba | 7693199.675 |
| Tokyo | 19743510.48 |
| Kanagawa | 10968902.79 |
| Niigata | 3098700.25 |
| Toyama | 1321633.001 |
| Ishikawa | 1635347.993 |
| Fukui | 1004969.61 |
| Yamanashi | 940121.9928 |
| Nagano | 2419597.32 |
| Gifu | 2981353.946 |
| Shizuoka | 5301405.547 |
| Aichi | 10353342.56 |
| Mie | 2563374.3 |
| Shiga | 1890457.788 |
| Kyoto | 3657714.678 |
| Osaka | 12696633.31 |
| Hyogo | 7556661.666 |
| Nara | 1864843.06 |
| Wakayama | 1414288.555 |
| Tottori | 721358.2043 |
| Shimane | 925933.1115 |
| Okayama | 2858356.395 |
| Hiroshima | 4515425.834 |
| Yamaguchi | 2105584.261 |
| Tokushima | 1116272.881 |
| Kagawa | 1536743.643 |
| Ehime | 2190657.764 |
| Kochi | 1054459.478 |
| Fukuoka | 7269763.737 |
| Saga | 1306235.988 |
| Nagasaki | 2016336.034 |
| Kumamoto | 2815633.421 |
| Oita | 1948135.59 |
| Miyazaki | 1639627.539 |
| Kagoshima | 2376539.142 |
| Okinawa | 1766863.046 |
| Secondary Medical Areas | |
| Minamioshima | 553963.0315 |
| Minamihiyama | 18633.59301 |
| Kitaoshimahiyama | 32322.78021 |
| Sapporo | 3051118.14 |
| Shiribeshi | 239029.1272 |
| Minamisorachi | 188107.4334 |
| Nakasorachi | 110821.5923 |
| Kitasorachi | 26016.40954 |
| Nishiiburi | 244410.9787 |
| Higashiiburi | 264727.3352 |
| Hidaka | 67610.59763 |
| Kamikawa-chubu | 544791.8974 |
| Kamikawa-hokubu | 68641.44307 |
| Hurano | 38100.91899 |
| Rumoi | 42498.27207 |
| Souya | 59056.03664 |
| Hokumou | 275785.7587 |
| Enmon | 45908.02524 |
| Tokachi | 398240.9118 |
| Kushiro | 288566.6219 |
| Nemuro | 68808.34988 |
| Tsugaru-Chiiki | 435277.9619 |
| Hachinohe-Chiiki | 399211.3871 |
| Aomori-Chiiki | 414142.1816 |
| Seihokugo-Chiiki | 136077.096 |
| Kamitousan-Chiiki | 184929.72 |
| Shimokita-Chiiki | 82956.716 |
| Morioka | 636640.1311 |
| Iwate-chubu | 207735.3153 |
| Tankou | 128528.9472 |
| Ryoban | 126617.8325 |
| Kesen | 59756.53687 |
| Kamaichi | 53203.52452 |
| Miyako | 70676.95654 |
| Kuji | 50906.97762 |
| Ninohe | 50750.68915 |
| Sennan | 172803.8944 |
| Sendai | 1948578.337 |
| Osaki, Kurihara | 318358.732 |
| Ishinomaki, Tomeshi, Kesennuma | 387096.9095 |
| Odate, Kaduno | 117104.8109 |
| Kitaakita | 38970.55763 |
| Noshiro, Yamamoto | 101057.8786 |
| Akita-Shuhen | 568094.1109 |
| Yurihonjou, Nikaho | 136988.6629 |
| Daisen, Senboku | 138308.4929 |
| Yokote | 158763.0248 |
| Yuzawa, Ogatsu | 50735.00381 |
| Murayama | 721226.024 |
| Mogami | 85901.12613 |
| Okitama | 230790.3111 |
| Shonai | 314751.3444 |
| Kenhoku | 564616.5906 |
| Kennaka | 687111.8093 |
| Kennan | 143677.3677 |
| Aizu | 349590.8255 |
| Minamiaizu | 28611.4927 |
| Soso | 134028.5131 |
| Iwaki | 484596.3839 |
| Mito | 709255.4109 |
| Hitachi | 274951.8958 |
| Hitachioota, Hitachinaka | 392811.0783 |
| Rokkou | 258100.7996 |
| Tsuchiura | 377930.7949 |
| Tsukuba | 538223.5522 |
| Toride, Ryugasaki | 564241.69 |
| Chikusei, Simotsuma | 252373.9134 |
| Koga, Bandou | 216505.9508 |
| Kenhoku | 377218.1695 |
| Kensei | 166891.0954 |
| Utsunomiya | 673591.2938 |
| Kentou | 128412.6458 |
| Kennan | 729480.9116 |
| Ryomou | 331894.1698 |
| Maebashi | 520518.6565 |
| Shibukawa | 532786.3397 |
| Isezaki | 126092.7109 |
| Takasaki, Annaka | 120031.9963 |
| Fujioka | 80230.24894 |
| Tomioka | 42979.31448 |
| Agatuma | 78209.49492 |
| Numata | 240790.877 |
| Kiryu | 165421.4739 |
| Ota, Tatebayashi | 467115.5859 |
| Nanbu | 908382.7078 |
| Nanseibu | 663783.6633 |
| Toubu | 1321943.838 |
| Saitama | 1494596.525 |
| Kenou | 531655.6091 |
| Kawagoehiki | 997732.3792 |
| Seibu | 761447.8988 |
| Tone | 671292.6466 |
| Hokubu | 589368.6376 |
| Chichibu | 109447.2987 |
| Chiba | 1366477.18 |
| Toukatsu-nanbu | 2051753.9 |
| Toukatsu-hokubu | 1684976.159 |
| Inba | 857581.2463 |
| Katorikaisou | 359115.9881 |
| Sanmushichouseiisumi | 432698.72 |
| Awa | 234408.2686 |
| Kimitsu | 339961.4837 |
| Ichihara | 366226.7291 |
| Ku-chuoubu | 3243134.704 |
| Ku-nanbu | 1564995.187 |
| Ku-seinanbu | 2032541.965 |
| Ku-seibu | 2078851.767 |
| Ku-seihokubu | 2456646.938 |
| Ku-touhokubu | 1565818.366 |
| Ku-toubu | 1824244.112 |
| Nishitama | 388929.195 |
| Minamitama | 1606580.475 |
| Kitatama-seibu | 713640.0964 |
| Kitatama-nanbu | 1321545.511 |
| Kitatama-hokubu | 929204.0733 |
| Tousyo | 17378.09075 |
| Yokohama-hokubu | 1842208.052 |
| Yokohama-seibu | 1322851.139 |
| Yokohama-nanbu | 1446317.422 |
| Kawasaki-hokubu | 918834.721 |
| Kawasaki-nanbu | 812772.1902 |
| Yokosuka, Miura | 936773.993 |
| Shonan-toubu | 896980.4957 |
| Shonan-seibu | 738089.7428 |
| Kenou | 871408.2092 |
| Sagamihara | 828418.4621 |
| Kensei | 354248.3646 |
| Kaetsu | 273581.3731 |
| Niigata | 1346580.833 |
| Kenou | 277415.3376 |
| Chuetsu | 637516.3308 |
| Uonuma | 163790.5423 |
| Jouetsu | 334086.4561 |
| Sado | 65729.37694 |
| Nikawa | 180201.2944 |
| Toyama | 633194.1363 |
| Takaoka | 355351.9938 |
| Tonami | 152885.5761 |
| Minamikaga | 309368.5628 |
| Ishikawa-chuou | 1094645.388 |
| Noto-chubu | 153393.9546 |
| Noto-hokubu | 77940.08774 |
| Fukui, Sakai | 590805.3121 |
| Okuetsu | 47528.54731 |
| Tannan | 183500.157 |
| Reinan | 183135.5933 |
| Chuhoku | 571138.5188 |
| Kyoutou | 138628.3337 |
| Kyonan | 47313.26326 |
| Fuji, Toubu | 183041.8771 |
| Saku | 257628.4461 |
| Kamiko | 262523.5404 |
| Suwa | 250641.2721 |
| Kamiina | 173055.7893 |
| Hanni | 154896.5255 |
| Kiso | 20110.36066 |
| Matsumoto | 552546.5185 |
| Taihoku | 57532.064 |
| Nagano | 594061.2117 |
| Hokushin | 96601.59132 |
| Gifu | 1423079.539 |
| Seinou | 478820.3123 |
| Chunou | 454800.4086 |
| Tounou | 452053.5449 |
| Hida | 172600.1415 |
| Kamo | 57716.5929 |
| Atamiitou | 137503.3429 |
| Suntoutagata | 925416.3176 |
| Fuji | 484918.0104 |
| Shizuoka | 1041313.032 |
| Shidahaibara | 649576.1477 |
| Chutouen | 688701.4089 |
| Seibu | 1316260.695 |
| Nagoya | 3626567.623 |
| Ama | 488733.466 |
| Owari-chubu | 189258.5531 |
| Owari-toubu | 820950.6347 |
| Owari-seibu | 668127.8127 |
| Owari-hokubu | 1012140.19 |
| Chitahantou | 733087.2584 |
| Nishimikawa-hokubu | 547640.8508 |
| Nishimikawa-nanbunishi | 877083.2117 |
| Nishimikawa-nanbuhigashi | 464564.684 |
| Higashimikawa-hokubu | 50479.81033 |
| Higashimikawa-nanbu | 874708.4634 |
| Hokusei | 1122177.421 |
| Chusei-iga | 641842.8912 |
| Nansei-shima | 715145.1038 |
| Higashikishuu | 84208.88417 |
| Otsu | 493388.4902 |
| Konan | 474011.1913 |
| Kouga | 166005.4422 |
| Higashioumi | 272924.768 |
| Kotou | 204961.3348 |
| Kohoku | 202156.7405 |
| Kosai | 77009.82075 |
| Tango | 88643.33613 |
| Chutan | 231648.3155 |
| Nantan | 131506.5334 |
| Kyoto, Otokuni | 2530660.288 |
| Yamashiro-kita | 517112.5844 |
| Yamashiro-minami | 158143.6205 |
| Toyono | 1340658.674 |
| Mishima | 1078534.204 |
| Kitakawachi | 1544104.543 |
| Nakakawachi | 928477.3971 |
| Minamikawachi | 893207.9761 |
| Sakai-shi | 1058911.397 |
| Senshu | 1251308.321 |
| Osaka-shi | 4601430.801 |
| Kobe | 2384923.895 |
| Hanshin-minami | 1486264.423 |
| Hanshin-kita | 834360.653 |
| Higashiharima | 874094.205 |
| Kitaharima | 340101.8468 |
| Nakaharima | 789054.5089 |
| Nishiharima | 328093.1013 |
| Tajima | 209738.9383 |
| Tanba | 123153.0148 |
| Awaji | 186877.0797 |
| Nara | 500930.3115 |
| Touwa | 338843.8029 |
| Seiwa | 449670.8978 |
| Chuwa | 512687.7855 |
| Nanwa | 62710.26225 |
| Wakayama | 704366.3404 |
| Naga | 166851.0795 |
| Hashimoto | 125602.5197 |
| Arita | 74899.08799 |
| Gobou | 75783.2648 |
| Tanabe | 169852.3666 |
| Shinguu | 96933.89582 |
| Toubu | 301019.6343 |
| Chubu | 116812.2799 |
| Seibu | 303526.29 |
| Matsue | 340228.2664 |
| Unnan | 51518.72591 |
| Izumo | 285357.7941 |
| Ota | 53695.77608 |
| Hamada | 107092.0526 |
| Masuda | 67093.89116 |
| Oki | 20946.60517 |
| Kennan-toubu | 1402963.514 |
| Kennan-seibu | 1057797.339 |
| Takahashi, Niimi | 57821.49222 |
| Maniwa | 66001.01482 |
| Tsuyama, Aida | 273773.0353 |
| Hiroshima | 2140825.327 |
| Hiroshima-nishi | 218751.495 |
| Kure | 412727.4448 |
| Hiroshima-chuou | 392989.3975 |
| Bisan | 382996.3397 |
| Fukuyama, fuchu | 842269.9939 |
| Bihoku | 124865.8361 |
| Iwakuni | 209381.6435 |
| Yanai | 93323.19303 |
| Shunan | 414907.3645 |
| Yamaguchi, Houhu | 462277.5884 |
| Ube, Onoda | 457440.4334 |
| Shimonoseki | 354984.0875 |
| Nagato | 50325.7712 |
| Hagi | 62944.17914 |
| Toubu | 833599.7369 |
| Nanbu | 186162.7921 |
| Seibu | 96510.35173 |
| Okawa | 107085.8337 |
| Syozu | 25670.25755 |
| Takamatsu | 750613.6228 |
| Chusan | 479028.4675 |
| Mitoyo | 174345.4613 |
| Uma | 118186.8825 |
| Nihama, Saijo | 322290.066 |
| Imabari | 244239.7407 |
| Matsuyama | 1120565.971 |
| Yawatahama, Ozu | 217367.5644 |
| Uwajima | 168007.54 |
| Aki | 62935.88779 |
| Chuou | 850909.1656 |
| Takahata | 43089.39609 |
| Hata | 97525.02869 |
| Fukuoka, Itoshima | 2385255.792 |
| Kasuya | 330713.4278 |
| Munakata | 249457.6463 |
| Tsukushi | 520099.1477 |
| Asakura | 109557.2257 |
| Kurume | 672887.5278 |
| Yame, Chikugo | 170185.6567 |
| Ariake | 312873.4562 |
| Iizuka | 231430.5327 |
| Noogata, Kurate | 146121.515 |
| Tagawa | 143521.4097 |
| Kitakyushu | 1777447.405 |
| Keichiku | 220212.994 |
| Chubu | 598063.3898 |
| Toubu | 171522.8273 |
| Hokubu | 208147.2043 |
| Seibu | 81654.68151 |
| Nanbu | 246847.8855 |
| Ngasaki | 836244.8317 |
| Sasebo-kenhoku | 467368.6373 |
| Kennou | 403749.9516 |
| Kennan | 170063.6301 |
| Gotou | 53370.10083 |
| Kamigotou | 25625.81071 |
| Iki | 31080.63563 |
| Tsushima | 28832.43668 |
| Kumamoto | 1357206.551 |
| Uki | 185963.21 |
| Ariake | 217104.7671 |
| Kamoto | 76613.33165 |
| Kikuchi | 231041.5221 |
| Aso | 56732.64702 |
| Kamimashiki | 133101.8759 |
| Yatsushiro | 188491.4355 |
| Ashikita | 75895.28955 |
| Kuma | 135776.9685 |
| Amakusa | 157705.8226 |
| Toubu | 343679.7131 |
| Chubu | 1026939.254 |
| Nanbu | 117746.2654 |
| Houhi | 83301.11453 |
| Seibu | 106544.1686 |
| Hokubu | 269925.0748 |
| Miyazaki-higashimorokata | 755725.338 |
| Miyakonojou-kitamorokata | 276839.6691 |
| Nobeoka-nishiusuki | 180835.9566 |
| Nichinankushima | 86282.32566 |
| Nishimoro | 130402.1905 |
| Saitokoyu | 93155.12192 |
| Hyugairigou | 116386.9375 |
| Kagoshima | 1124697.406 |
| Nansatsu | 193623.3942 |
| Sensatsu | 157776.7251 |
| Izumi | 96599.24556 |
| Aira, Isa | 344774.4639 |
| Soo | 87502.7616 |
| Kimotsuki | 218162.1291 |
| Kumage | 42699.31465 |
| Amami | 110703.7026 |
| Hokubu | 114980.3705 |
| Chubu | 499151.0524 |
| Nanbu | 1046168.489 |
| Miyako | 55640.85062 |
| Yaeyama | 50922.28332 |
