# Supplementary material for: Effect of population inflow and outflow between rural and urban areas on regional antimicrobial use surveillance
Source: PLoS One. 2021 Mar 18;16(3):e0248338. doi: 10.1371/journal.pone.0248338 (PMC7971456; doi:10.1371/journal.pone.0248338)
Supplement: Attachment — Submitted filename: PONE-D-20-15330_R1_Response_to_Editors_and_Reviewers.docx [file pone.0248338.s004.docx]

**Response to Editors**

Thank you for your advice and positive evaluation of our manuscript. We have revised the manuscript and online submission form in accordance with your instructions. Our point-to-point responses are provided below.

1. Please ensure that your manuscript meets PLOS ONE's style requirements, including those for file naming.

Thank you for your advice. We have ensured that the manuscript meets PLOS ONE’s style requirements, and corrected the file names according to the author guidelines.

2. In your ethics statement in the Methods section and in the online submission form, please clarify whether all data were fully anonymized before you accessed them.

The data were fully anonymized before being received by the authors. As instructed, we have added this statement to the Methods section and in the online submission form.

Page 11, Lines 176-177

The study did not involve any interventions in human subjects, and the NDB data were anonymized before being received by the authors.

3. Please state the date(s) when the data was obtained/accessed from the NDB.

The data were accessed in January 2020. We have added this statement to the manuscript.

Page 7, Lines 118-119

The NDB data were accessed in January 2020.

4. Thank you for stating in your manuscript text. "This study was approved by the institutional review board of the National Center for Global Health and Medicine (Approval Number: NCGM-G-002505-00)." Please also include this in your ethics statement in the online submission form.

We have included this sentence in the ethics statement in the online submission form.

5. Please include a table of relevant demographic details summarizing the studied population.

As advised, we have created a new supplementary table (S2 Table) that shows the demographic details of the populations used in the analysis.

6. Please upload a copy of Figure 4, to which you refer in your text on page 16. If the figure is no longer to be included as part of the submission please remove all reference to it within the text.

We sincerely apologize for omitting Figure 4, and have included it in this resubmission.

7. Please include captions for your Supporting Information files at the end of your manuscript, and update any in-text citations to match accordingly.

Thank you for pointing this out. We have included the supplementary table captions at the end of the manuscript.

**Response to Reviewers**

Reviewer #1:

Response: Thank you for your time and effort in reviewing our manuscript. We have revised the manuscript in accordance with your suggestions.

We have added a statistical analysis of the differences between the national-level DID values and the prefectural- and SMA-level DID values. This analysis showed that the SMA-level DID was significantly different from the national-level DID, irrespective of whether standardization was performed with the nighttime or daytime population. Therefore, as a major revision, we have softened the tone of our conclusion that we should use the daytime standardized–population as the SMA-level AMU’s denominator, and indicated that it may be better to avoid using SMA-level DID. Finally, we apologize for omitting Figure 4, and have included it in this resubmission. Thank you for giving us the chance to revise our manuscript, and we look forward to your evaluation.

Conclusions (Page 20, Lines 334-338)

Before

AMU surveillance has conventionally used the nighttime population for standardization. However, it is evident that nighttime population standardization can lead to erroneous estimates due to its omission of population inflow and outflow, especially for smaller regions. In Japan, the estimation of SMA-level DID values should be based on daytime population standardization to enable more impartial comparisons.

After

AMU surveillance has conventionally used the nighttime population for standardization. However, regional-level AMU estimates, especially of smaller regions such as SMAs, are more susceptible to the influence of whatever population is used for standardization, which can lead to erroneous estimates. Nevertheless, daytime populations that account for population inflow and outflow may be preferable to nighttime populations to minimize such errors and enable more precise comparisons.

1. The discussion is concise, but would benefit from a further elaboration of how this recommended shift from nighttime to daytime population usage will change antimicrobial use policy and management. What implications does this have for future regulation? What steps will be taken next? A more in-depth discussion would be helpful.

Thank you for your advice. As suggested, we have addressed the potential implications of our findings, although we prefer to refrain from a more in-depth discussion at this stage (we foresee that the shift from nighttime to daytime population could help produce more accurate AMU estimates, but anything downstream of that would be speculative). In this study, we identified population in/out flow to be one of the factors that can introduce bias into regional AMU estimates. As a next step, we aim to further elucidate the other factors that affect regional AMU, and to develop a standardization formula similar to the Standardized Antimicrobial Administration Ratio, which was developed by the US CDC and is used in hospital AMU comparisons. We have added this perspective to the end of the Discussion section.

Page 20, Lines 322-331

The shift from nighttime population to daytime population for standardization could provide more accurate regional-level AMU estimates, which would help to inform AMU-related policymaking, guide the efficient allocation of resources for region-specific antimicrobial stewardship programs, and contextualize the decision-making process for AMU regulations. As a next step, we aim to develop a formula to standardize regional-level AMU that can account for various sources of biases (e.g., population inflow and outflow) similar to the Standardized Antimicrobial Administration Ratio, which was developed by the US Centers for Disease Control and Prevention and is used in hospital-level AMU comparisons. In this way, our study represents the first step for developing a standardization method for regional-level AMU surveillance in Japan. Further studies are needed to identify the other factors of regional AMU surveillance.

Reference

14.	van Santen K L, Edwards JR, Webb A K, Pollack L A, O’Leary E, Neuhauser M N, et al. The Standardized Antimicrobial Administration Ratio: A New Metric for Measuring and Comparing Antibiotic Use. Clin Infect Dis. 2018;67: 179-185. doi: 10.1093/cid/ciy075

2. I did not receive Figure 4 – the description is provided, but not the figure itself. This should be provided and reviewed.

We apologize for omitting Figure 4. This figure has been included in the resubmitted manuscript, and we would appreciate any comments or advice.

3. Figure 3 displays at a quite small size, making the color of the map and differences between region SMA difficult to see. Would enlarge.

Thank you for pointing this out. We have reformatted Figure 3 with a higher resolution (1200 dpi) and enlarged. We hope that it is more readable.

Reviewer #2:

Response: Thank you for your time and effort in reviewing our manuscript. We have revised the manuscript in accordance with your suggestions.

We have added a statistical analysis of the differences between the national-level DID values and the prefectural- and SMA-level DID values. This analysis showed that the SMA-level DID was significantly different from the national-level DID, irrespective of whether standardization was performed with the nighttime or daytime population. Therefore, as a major revision, we have softened the tone of our conclusion that we should use the daytime standardized–population as the SMA-level AMU’s denominator, and indicated that it may be better to avoid using SMA-level DID. Finally, we apologize for omitting Figure 4, and have included it in this resubmission. Thank you for giving us the chance to revise our manuscript, and we look forward to your evaluation.

Conclusions (Page 20, Lines 334-338)

Before

AMU surveillance has conventionally used the nighttime population for standardization. However, it is evident that nighttime population standardization can lead to erroneous estimates due to its omission of population inflow and outflow, especially for smaller regions. In Japan, the estimation of SMA-level DID values should be based on daytime population standardization to enable more impartial comparisons.

After

AMU surveillance has conventionally used the nighttime population for standardization. However, regional-level AMU estimates, especially of smaller regions such as SMAs, are more susceptible to the influence of whatever population is used for standardization, which can lead to erroneous estimates. Nevertheless, daytime populations that account for population inflow and outflow may be preferable to nighttime populations to minimize such errors and enable more precise comparisons.

1. The authors don’t’ compare median value of DID standardized between nighttime and daytime population. Are the differences statistically significant?

Thank you for your important advice. We have consulted with a statistician regarding this concern. Because prefectural- and SMA-level DID values do not have a “correct value” or gold standard, it would be difficult to interpret a statistically significant difference in median values between the nighttime and daytime populations. Instead, we have calculated the correlations between daytime and nighttime population–standardized DID in Figure 2. Furthermore, in Figure 1, we show the mean and 95% confidence interval for each indicator, and created violin plots to visualize their dispersions. Additionally, we compared each DID value to the national-level AMU using the one-sample *t*-test. The results showed that neither the daytime nor nighttime population–standardized DID at the prefectural level had any significant difference with the national-level AMU. However, both the daytime and nighttime population–standardized DID values at the SMA level were significantly different from the national-level AMU. Accordingly, we concluded that SMA-level DID values standardized using either of these populations would be difficult to evaluate. However, the 95% confidence interval was narrower and the mean (center of the widest section in the violin plot) was closer to the national AMU in the daytime population–standardized DID than in the nighttime population–standardized DID at the SMA level. Therefore, if SMA-level DID values must be calculated, the daytime population may represent the better option for standardization. We have added these points to the manuscript as follows:

Abstract (Page 3, Lines 38-42)

The national AMU was 17.21 DDDs per 1,000 population per day. The mean (95% confidence interval) prefectural-level DDDs per 1,000 nighttime and daytime population per day were 17.27 (14.10, 20.44) and 17.41 (14.30, 20.53), respectively. The mean (95% confidence interval) SMA-level DDDs per 1,000 nighttime and daytime population per day were 16.12 (9.84, 22.41) and 16.41 (10.57, 22.26), respectively.

Methods (Page 9, Lines 147 -150)

Next, we evaluated the distributions of daytime and nighttime population–standardized DID values at the prefectural and SMA levels using the Kolmogorov–Smirnov test. The mean DID values were compared with the national AMU using the one-sample *t*-test.

Results (Page 11, Lines 183-197)

When standardized with the nighttime population, the mean DID was 17.27 (95% confidence interval [14.10, 20.44]) at the prefectural level and 16.12 (95% confidence interval [9.84, 22.41]) at the SMA level. When standardized with the daytime population, the median DID was 17.41 (95% confidence interval [14.30, 20.53]) at the prefectural level and 16.41 (95% confidence interval [10.57, 22.26]) at the SMA level. Both the prefectural-level and SMA-level DID values were normally distributed regardless of the population used for standardization. The daytime and nighttime population–standardized mean DID values at the prefectural level were not significantly different from the national-level DID values (nighttime: *P* = .385; daytime: *P* = .811); however, the corresponding mean DID values at the SMA level were significantly different from the national-level DID values (nighttime: *P* < .001; daytime: *P* < .001). As shown in Fig 1, daytime population–standardized DID at the SMA level had a narrower dispersion and a mean value (center of the widest section in the violin plot) that was closer to the national-level DID than the nighttime population–standardized DID. In contrast, the daytime population and nighttime population–standardized DID at the prefectural level exhibited similar shapes in the violin plot.

Discussion (Page 17, Lines 269-279)

Previous studies have examined the effects of using different denominator values when calculating AMU in hospitals.^9–12^ However, these effects on regional AMU surveillance have not been explored. Even the World Health Organization’s AMU surveillance methodology does not address the appropriate methods for analyzing sub-national regions.^13^ In our analysis, SMA-level AMU (standardized using either the daytime or nighttime population) was found to be significantly different from the national-level AMU. This suggests that the population-standardized DID values of smaller regions are susceptible to the effects of population inflow and outflow, which can lead to erroneous results.

At the SMA level, daytime population–standardization produced fewer outliers, narrower 95% confidence intervals, and mean DID values that were closer to the national-level DID than nighttime population–standardization.

Discussion (Page 18, Lines 283-284)

Although standardization using either population may be problematic for SMAs, our findings indicate that the use of the daytime population is preferable to the nighttime population for such adjustments.

2. In Figure 1 I think that the crosses represent the median and not the mean value as described in legend of figure 1.

Thank you for the suggestion. As mentioned in the response to your previous comment, we have replaced the boxplots with violin plots.

3. I suggest to the authors to estimate an indicator of agreement to evaluate the relationship between nighttime population–standardized DID values respect to daytime population–standardized DID values. (figure 2)

As advised, we calculated Pearson’s correlation coefficients and *P* values for Figure 2. The following sentences have been added:

Methods (Page 9, Lines 152-153)

Correlations between nighttime and daytime population–standardized DID values were examined using Pearson’s correlation coefficients.

Results (Page 12, Lines 205-207)

The correlation coefficient between nighttime and daytime population–standardized DID values was higher at the prefectural level (0.90; *P* < .001) than at the SMA level (0.80; *P* < .001).

4. Figure 4 is not included in the submission material

We apologize for omitting Figure 4. This figure has been included in the resubmitted manuscript, and we would appreciate any comments or advice.
