# Supplementary material for: Effect of population inflow and outflow between rural and urban areas on regional antimicrobial use surveillance
Source: PLoS One. 2021 Mar 18;16(3):e0248338. doi: 10.1371/journal.pone.0248338 (PMC7971456; doi:10.1371/journal.pone.0248338)
Supplement: Attachment — Submitted filename: PONE-D-20-15330_R2_Response_to_reviewer.docx [file pone.0248338.s005.docx]

**Response to Reviewers**

Reviewer #3:

Thank you for your useful advice. We have corrected our manuscript according to your suggestions. We hope that our revisions meet your requirements.

Major Comments:

The authors make claims about which population value (nighttime vs. daytime) should be used to calculate AMU, based on which is more correct. However, the datasets used include no information about the "true" AMU, so it is unclear to me what this claim in based on. The authors do rightly make claims as to where this difference in calculation could lead to the widest discrepancies between the two metrics, but I don't understand how this leads to the conclusion that it is better to use daytime populations. Perhaps there is a well-supported assumption that people are prescribed medicine near their work and school places, but I am not familiar with the Japanese healthcare system and so do not know if this is the case. Other readers may also not be familiar with Japanese healthcare, and it would be helpful if the authors could provide more reasoning for the case they make.

Thank you for pointing this out. We agree with your opinion. Although we described that daytime population standardization is a superior method for estimating AMU in small regions than nighttime population standardization, this is not justified because of the absence of a standard AMU, as you pointed out. Therefore, we have corrected the Discussion and Conclusions as follows:

Abstract (Lines 47-49)

Before

Regional-level AMU estimates, especially of smaller regions such as SMAs, are susceptible to the use of different populations for standardization. Daytime populations that account for population in/outflow may be preferable to nighttime populations for such adjustments.

After

Regional-level AMU estimates, especially of smaller regions such as SMAs, are susceptible to the use of different populations for standardization. This finding indicates that AMU standardization based on population values is not suitable for AMU estimates in small regions.

Discussion (Line 278-285)

Before

At the SMA level, daytime population–standardization produced fewer outliers, narrower 95% confidence intervals, and mean DID values that were closer to the national-level DID than nighttime population–standardization. These findings showed that when analyzing smaller regional units such as SMAs, the use of different populations for standardization had a considerable effect on DID estimates in central urban areas and their surrounding regions. As more medical examinations and prescriptions are received during the day than at night, the calculation of regional AMU indices should account for the effects of population inflow and outflow. Although standardization using either population may be problematic for SMAs, our findings indicate that the use of the daytime population is preferable to the nighttime population for such adjustments.

After

As more medical examinations and prescriptions are received during the day than at night, the calculation of regional AMU indices should account for the effects of population inflow and outflow. At the SMA level, daytime population standardization produced fewer outliers, narrower 95% confidence intervals, and mean DID values that were closer to the national DID than nighttime population standardization. These findings showed that when analyzing smaller regional units, such as SMAs, the use of different populations for standardization has a considerable effect on DID estimates in central urban areas and their surrounding regions. Our insights indicate that AMU standardization based on population values is not suitable for AMU estimates in small regions.

Discussion (Line 320-323)

Before

The shift from nighttime population to daytime population for standardization could provide more accurate regional-level AMU estimates, which would help to inform AMU-related policymaking, guide the efficient allocation of resources for region-specific antimicrobial stewardship programs, and contextualize the decision-making process for AMU regulations. As a next step, we aim to develop a formula to standardize regional-level AMU that can account for various sources of biases (e.g., population inflow and outflow) similar to the Standardized Antimicrobial Administration Ratio, which was developed by the US Centers for Disease Control and Prevention and is used in hospital-level AMU comparisons. In this way, our study represents the first step for developing a standardization method for regional-level AMU surveillance in Japan. Further studies are needed to identify the other factors of regional AMU surveillance.

After

Although regional AMU estimates would help inform AMU-related policymaking, we recommend avoiding AMU evaluation in small regions standardized by the population number. If we wish to monitor AMU evaluations in small regions, only the temporal change in a region should be considered, for example.

Conclusion (Line 329-330)

Before

Nevertheless, daytime populations that account for population inflow and outflow may be preferable to nighttime populations to minimize such errors and enable more precise comparisons.

After

Therefore, new approaches are required to monitor AMU evaluations in small regions, for example, observing only the temporal changes in a region.

Minor Comments:

1. Line 162: Do you mean the populations size of each age category for each SMA?

Yes, we used population numbers according to the age categories. We have corrected the description as you indicated.

Methods (Line 161-162)

Before

Finally, we divided each SMA’s population into three categories based on age (children: <15 years, working-age adults: 15–64 years, and older adults: ≥65 years), and analyzed the correlation between the absolute difference in DID values and each age category. For this analysis, the Tokyo/Ku-chuoubu SMA was again excluded as it was an extreme outlier.

After

Finally, we used the population number for each age category (children: <15 years, working-age adults: 15–64 years, and older adults: ≥65 years) for each SMA, and analyzed the correlation between the absolute difference in DID values and each age category. For this analysis, the Tokyo/Ku-chuoubu SMA was excluded as it was an extreme outlier.

2. Line 186: Why do you compare median here and mean on line 184? Is one of these a typo, since you do compare them later in this paragraph?

Thank you for pointing this out. We corrected “median” to “mean.”

3. Table 1: What do the three different tables in Table 1 represent? Is it possible to add a sub-title, such as a row that seperates each table that lables what each one is?

We added descriptions to each table.

Result, Table.1 (Lines 251)

**Table 1. Difference between nighttime population–standardized and daytime population–standardized defined daily doses per 1,000 population per day**

| Rank | Prefecture/Secondary medical area | Difference |
| --- | --- | --- |
| Difference in AMU between nighttime population-standardized and daytime population–standardized (ranked in descending order) | | |
| 1 | Tokyo/Ku-chuoubu | 30.94 |
| 2 | Osaka/Osaka-shi | 4.94 |
| 3 | Tokyo/Ku-seibu | 4.50 |
| 4 | Tokyo/Ku-seinanbu | 3.15 |
| 5 | Fukushima/Soso | 2.30 |
| 6 | Aichi/Nagoya | 2.24 |
| 7 | Tokyo/Ku-nanbu | 1.99 |
| 8 | Fukuoka/Itoshima | 1.49 |
| 9 | Fukuoka/Noogata, Kurate | 1.34 |
| 10 | Aichi/Nishimikawa-hokubu | 1.25 |
| Difference in AMU between nighttime population-standardized and daytime population-standardized (ranked in ascending order) | | |
| 1 | Kyoto/Yamashiro-minami | -4.42 |
| 2 | Tokyo/Kitatama-hokubu | -3.78 |
| 3 | Fukuoka/Munakata | -3.70 |
| 4 | Kanagawa/Kawasaki-hokubu | -3.69 |
| 5 | Wakayama/Naga | -3.28 |
| 6 | Saitama/Nanbu | -2.91 |
| 7 | Aichi/Ama | -2.86 |
| 8 | Saitama/Keno | -2.85 |
| 9 | Nara/Seiwa | -2.82 |
| 10 | Chiba/Toukatsu-hokubu | -2.82 |
| Absolute value of the AMU difference between nighttime population-standardized and daytime population-standardized (ranked in ascending order) | | |
| 1 | Shimane/Masuda | 0.00 |
| 2 | Hokkaido/Tokachi | 0.00 |
| 3 | Yamagata/Murayama | 0.00 |
| 4 | Hokkaido/Kushiro | 0.00 |
| 5 | Gifu/Hida | 0.01 |
| 6 | Fukushima/Kennaka | 0.01 |
| 7 | Kouchi/Chuou | 0.01 |
| 8 | Kagoshima/Nansatsu | 0.01 |
| 9 | Kagoshima/Amami | 0.01 |
| 10 | Ehime/Matsuyama | 0.01 |

4. Line 284: It is not clear to me why the daytime-population standardized DID is better (see comment above). Could you explain this further?

Thank you. We corrected our discussion and conclusions as presented above.

5. Fig. 3: Please label the color bar with what it represents.

Thank you. We have added explanations of the color bars.

6. The authors have made the population data available in the supplement, but the data on AMU is not available, which is required to conduct the analyses.

Thank you. We have created Supplementary Table 3 to display AMU data.

Methods (Line 144-145)

National-level, prefectural-level, and SMA-level DDDs were shown in Table S3.
